# Integrating text mining, data mining, and network analysis for identifying genetic breast cancer trends

**DOI:** 10.1186/s13104-016-2023-5

**Published:** 2016-04-26

**Authors:** Gabriela Jurca, Omar Addam, Alper Aksac, Shang Gao, Tansel Özyer, Douglas Demetrick, Reda Alhajj

**Affiliations:** Department of Computer Science, University of Calgary, Calgary, AB Canada; College of Computer Science and Technology, Jilin University, Changchun, China; Department of Computer Engineering, TOBB University, Ankara, Turkey; Departments of Pathology, Oncology and Biochemistry & Molecular Biology, University of Calgary, Calgary, AB Canada; Department of Computer Science, Global University, Beirut, Lebanon

**Keywords:** Breast cancer, Data mining, Text mining, Network analysis

## Abstract

**Background:**

Breast cancer is a serious disease which affects many women and may lead to death. It has received considerable attention from the research community. Thus, biomedical researchers aim to find genetic biomarkers indicative of the disease. Novel biomarkers can be elucidated from the existing literature. However, the vast amount of scientific publications on breast cancer make this a daunting task. This paper presents a framework which investigates existing literature data for informative discoveries. It integrates text mining and social network analysis in order to identify new potential biomarkers for breast cancer.

**Results:**

We utilized PubMed for the testing. We investigated gene–gene interactions, as well as novel interactions such as gene-year, gene-country, and abstract-country to find out how the discoveries varied over time and how overlapping/diverse are the discoveries and the interest of various research groups in different countries.

**Conclusions:**

Interesting trends have been identified and discussed, e.g., different genes are highlighted in relationship to different countries though the various genes were found to share functionality. Some text analysis based results have been validated against results from other tools that predict gene–gene relations and gene functions.

**Electronic supplementary material:**

The online version of this article (doi:10.1186/s13104-016-2023-5) contains supplementary material, which is available to authorized users.

## Background

### Introduction

CANCER is one of the most serious and harmful diseases threatening humanity and may lead to death. Unfortunately there is no discovered robust treatment which leads to guaranteed cure from cancer. Thus, researchers from various domains are still working hard to identify molecules (mainly genes or proteins) which could be handled and targeted as cancer biomarkers. Various methods have been developed. The research spans a wide range of techniques from wet-lab testing by biologists to computational methods by computer scientists. The latter research is promising because it helps in tremendously reducing the number of molecules to consider as potential biomarkers.

Cancer is a result of damage (mutation) to a cell’s DNA (deoxyribonucleic acid), so that the cell loses normal functionality and instead gains the ability to indefinitely multiply until normal tissue functions are impaired [1]. Cancerous DNA mutations may occur from a complex mixture of inherited and external (environmental) factors, where these mutations are usually located in cell division genes [1]. There are over 100 known different types of cancer, depending on the cell type which was originally affected [1]. Additionally, each patient may have a different set of cancerous mutations in various genes, which may lead to different subtypes of the cancer. In order to personalize therapeutic strategies for cancer patients, medical researchers aim to identify and characterize the biomarkers of each type of cancer, so that they can provide the most accurate diagnosis to patients [2]. A cancer biomarker refers to a substance or process that serves as indication of cancer in the body, where one common example of a cancer biomarker is genetics [3].

The basic unit of genetic biomarkers are genes. A gene is one unit of the DNA which often contains the information needed to produce proteins. The central dogma is that genes are transcribed into an intermediate molecules called RNA, and the RNA is then translated into proteins, where proteins carry out the basic functions of life [4]. If a gene codes for a protein whose function is to suppress cancer, then if that gene is damaged or is downregulated (not transcribed enough), then the cell may become cancerous. Similarly, if a gene codes for a protein whose function is to promote cancer, then if that gene is upregulated (transcribed more than usual), then that cell may also become cancerous. Therefore, finding the different genes and conditions which are likely to lead to cancer, should the genes be upregulated or downregulated, is an important task for characterizing types of cancer. The problem is not trivial because there are various internal and external factors that might affect the cells leading to cancer. People do not have the same habits and behavior. Thus they may develop the same cancer differently based on the environment they live in, their diet, drinking, etc. Also, some types of cancer, such as breast and prostate cancer can be strongly influenced by inherited gene mutations, and often run in families [5]. Therefore, these heritable types of cancer may be predicted by examining a person’s DNA before they develop cancer. Identifying the heritable genetic mutations that increase the likelihood for cancer are critical to developing predictive genetic tests.

**Fig. 1:**
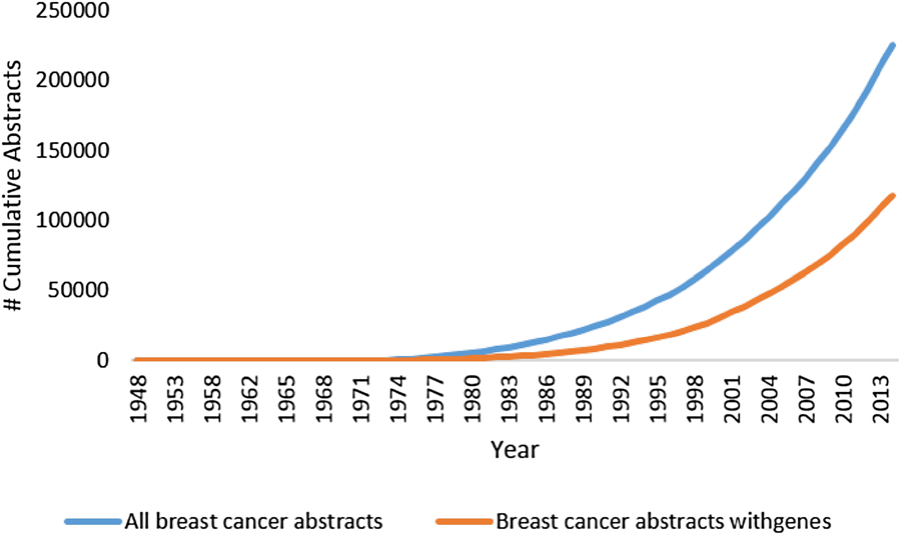
Growth in number of abstracts about breast cancer in PubMed

Our framework described in this paper is built on the hypothesis which could be articulated as follows. To investigate cancer biomarkers, one may investigate the literature which contains a huge amount of information hidden in the form of scientific articles. However, a query for “breast cancer” to PubMed can retrieve over 250,000 articles, which makes it impossible to get a full-picture of the field by reading them. The trend is that the number of PubMed articles are steadily increasing, and so are articles on the topic of breast cancer that mention gene names argued as potential biomarkers (see Fig. 1). Therefore, using text mining techniques to gather new knowledge from many existing scientific sources can be an effective way to investigate the literature for new biomarkers. One type of relationship which can be discovered is gene-disease, that shows which gene is involved in which disease [6]. Another type of relationship which can be found are gene–gene interactions [7].

Some data mining techniques that can be used to extract hidden information from a database are hard clustering, soft clustering, hierarchical clustering, and frequent pattern mining [8]. All of the aforementioned techniques are described in more detail in “Results and discussion” section. Each data mining technique utilizes different interestingness metrics, so it is useful to apply many techniques to a data set. Another technique we used on the genes extracted from the breast cancer abstracts was network analysis, or “Social Network Analysis” as it is sometimes referred to [9]. Network analysis has its roots in sociology, as it was first used to study the relationships and community structures in social data. However, network analysis has since been applied in other fields such as bioinformatics in order to find key molecular markers and communities within an interaction network.

To validate genes linked to cancer, one of the most effective ways is to analyze disease specific gene expression data [10].

Gene expression data is experimental data which can be used to check whether a gene has indeed been upregulated or downregulated with respect to a disease. This methodology compares to what level genes were expressed in cancerous cells versus healthy cells. It is unaffordable and infeasible to try wet-lab analysis of such a huge set of genes. Therefore, machine learning and data mining techniques (including frequent pattern mining, clustering and classification) can be used to lower this number of genes down to a manageable set of genes which are anticipated to be statistically linked with the disease. This way, biologists will concentrate only on the identified small set as potential cancer biomarkers instead of unrealistic case of testing every gene in the wet-lab as potential cancer biomarker. In other words, data mining techniques can save the time and cost of cancer researchers, turning their research goals into something potentially achievable. This is illustrated by the test results reported in this paper.

The paper is organized as the following sections. The problem explanation is made in “Problem explanation” section. “Related work” section describes the work related to our solution. In “The developed solution” section, the developed methodology is given in detail. The experimental results are depicted in “Evaluation of the developed solution” section. Lastly, contributions and future work are mentioned in “Results and discussion” section.

### Problem explanation

Identifying cancer biomarkers is not a trivial task. Despite all the effort, time, and money invested so far, the progress is still very little. Indeed the body is affected by various internal and external factors which altogether may lead to cancer. As the factors differ from person to person, the samples taken from two cancer patients may not reveal exactly the same information. Thus, there is a need to develop new techniques which could better analyze the existing sources of data with the hope to lead to more useful discoveries.

In this paper, we aimed to perform large-scale text analysis of biomedical abstracts in order to generate new hypothesis about cancer biomarkers. The target was to develop a data mining methodology, which would lead to patterns in the genes which are associated with cancer. In the this section we will discuss the tasks involved in text mining.

#### Text mining

Text mining is typically comprised of four stages [11, 12]: (1) information retrieval (IR), where a set of textual materials are gathered for a given topic; (2) entity recognition (NER), where textual features are identified from the gathered texts; (3) information extraction (IE) which aims to extract relationships among the recognized textual features; (4) knowledge discovery (KD), where the extracted relationships are used to identify useful patterns from the data set. The rest of this section is dedicated to explain each stage and how they can be applied to biomedical text mining.

##### Information retrieval for text mining

The first step in text mining is to gather the papers which are relevant to the topic of interest. There are a number of IR systems, including centralized institutional like PubMed and UK PubMed Central (UKPMC), or commercial systems like google scholar. The best known one is PubMed [11–13], which searches the MedLine database.

First, we can categorize an IR engine by the input. The topic may come from a query provided by the user, and this method of defining the topic is called ad hoc [14]. The other kind of IR system is called text categorization, where the input is a set of papers. Ad hoc has some limitations compared to text categorization [13]. PubMed is an ad-hoc system. Second, we can also categorize IR engines in terms of the scope of content delivered. For example, PubMed produces a comprehensive search of articles, but only retrieves the abstracts of the articles. In contrast, UKPMC returns the full text of articles [13].

##### Entity recognition (NER)

Once we have a subset of the available scientific literature which pertains to our topic, we must identify terms which are relevant to our study. NER has the aim of identifying terms within the gathered text, such as the names of different proteins or genes. The first task of these systems is to identify the biological entity names. The second task of NER is to identify the unique entity names. However, identifying biological terms is challenging due to the following reasons [12]:

- Biomedical terms often have synonyms (e.g., PTEN and MMAC1 refer to the same gene).
- A term may have different meanings (e.g., Cancer can also mean the astrological sign).
- Acronyms may lead to ambiguities (e.g., BC may mean breast cancer or it may mean British Columbia).

These challenges can make the naming of the biological entities quite imprecise. However, some strategies to overcome these drawbacks have been implemented in NER systems. One method is to integrate different vocabularies and ontologies which hold complete lists of biological entity names [12]. For example, gene ontology is a classification effort to describe what we know about genes, including to develop controlled vocabularies about those genes.

Early NER systems were rule-based with manually designed rules based on word structures. More recently NER systems have shifted to machine learning techniques which can recognize characteristics of words. A third type of NER systems is dictionary-based, which is the most effective due to the fact that it can recognize synonyms. In addition, it is also possible to use algorithms which can disambiguate acronyms automatically [11]. Some examples of NER systems that recognize biomedical entities are NCBO annotator, cTAKES, MetaMap, and BeCAS. A study which compared these four systems using their own ground truth determined that BeCAS performed differently compared to the other three systems [15]. BeCAS performed more poorly overall, but BeCAS recognized larger sentences than the other systems, which may have been underrepresented in their evaluation [15].

**Fig. 2:**
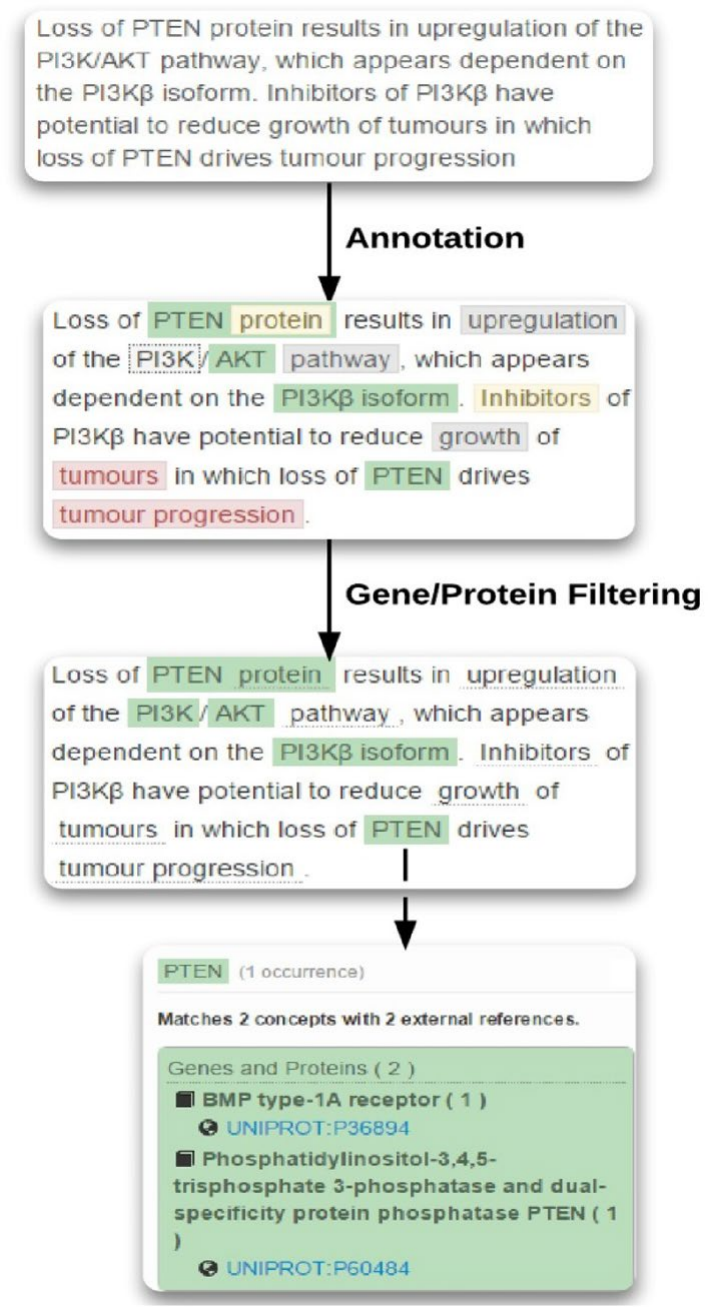
Text annotation using BeCAS

Figure 2 shows how an NER system may annotate biomedical terms. For example, in our problem, we require the genes associated with breast cancer. Therefore, we may use BeCAS to first find biomedical terms, then to label proteins and genes, followed by verification with the UniProt database using the given UniPROT ID. UniProt is a database which stores genes and proteins information.

##### Information extraction (IE)

The aim of IE is to extract relationships between the biological entities mentioned in the text. There are two approaches for this: co-occurrence processing and natural language processing (NLP) [11]. In co-occurrence processing, the entities are deemed to be related if they occur in the same text. For example, the relationships found are usually of the type gene–gene, or gene-disease. However, in co-occurrence processing, one cannot extract directional relationships between entities.

Through NLP, the directionality of the relationship between the biological entities can also be found. NLP analyzes the syntax and semantics of the sentence which contains the entities. However, NLP is better suited for full-text mining rather than abstract mining. The concise nature of abstracts makes it difficult to analyze the context of the biological entities [14]. Also, due to their complexity, NLP systems are designed for limited and specific types of relationships, and only a few systems can recognize multiple types of relationships [14]. As further discussed in “The developed solution” section, we used BeCAS API [16] to annotate and extract co-occurrences of biomedical concepts such as gene, protein, etc.

##### Knowledge discovery (KD)

KD is the extraction of knowledge from a large volume of structured and/or unstructured data. The goal of KD is to uncover novel knowledge from existing data. Novel data can be in the form of hidden relationships among biological entities. For example, if *A* is related to *B*, and *B* is related to *C*, text mining can infer the relationship that *A* is related to *C*. It is difficult for people to discover indirect relationships from a large amount of data. KD is often used to gain biologically meaningful knowledge about how biological entities are related.

#### Hypothesis generation

One of the newer approaches described in the literature is to generate scientific hypotheses through text mining [11, 13]. KD can be used to generate scientific hypotheses, for example about relationships between entities, which have yet to be validated. Whereas KD attempts to discover biological meaning about a set of facts, hypothesis generation attempts to discover whole new relationships. Hypothesis generation can be useful at directing scientists to which genes they should study without wasting much resources on the exploration.

The work described in [11] describes two ways in which hypothesis generation can occur: one way is to start with the microarray data to identify genes hypotheses, and then to support these hypotheses with literature mining. The second is to generate hypotheses through literature mining, and then validate the hypotheses through experimental data, such as microarray data. We decided to investigate the second method of hypothesis generation; actually, Faro et al. [11] identified the field as more lacking in research.

#### Evaluation

Some related work that use biomedical text mining to generate hypotheses have evaluated their results with experimental data [11, 17]. Experimental data can consist of gene expression data, which often comes in the form of microarray data. Gene microarray experiments are performed using specific tissue samples, and they measure the presence of the intermediate molecule RNA, so that we can know which genes are important in particular conditions [18]. Some genes may be up- regulated, which means that they were transcribed more, and we say that these genes were ‘expressed’. Otherwise, the genes may be down-regulated, which means that the genes were not ‘expressed’. Genes that were expressed together at the same time may have a relationship together, and we say they are ‘co-expressed’.

There are publicly available online repositories that store experimental data, as well as the gene–gene relationships and gene functionalities derived from experimental data. Some tools such as GeneMania have been built that show the relationships between genes by integrating information from various databases [19]. Tools such as GeneMania may be useful for validating the gene–gene relationship hypotheses. There are also tools such as DisGeNet [20, 21] and FunDo [22] that identify gene-disease relationships from curated sources.

### Related work

Faro et al. [11] described the methodology of hypothesis generation from literature, combined with experimental data evaluation, to be quite novel in 2011. In this section, we will describe some of the tools and methodologies which have been used for hypothesis generation from biomedical literature.

GeneWizard is an application which allows users to generate biological hypotheses based on text mining, and then evaluate the hypotheses through gene expression data [17]. One advantage of this tool is that it can be used to generate hypotheses about genes of any disease, whereas our methodology has so far been focused on breast cancer. However, in the future we aim to try our methodology on other cancer or diseases as well.

For the IR step, GeneWizard also used PubMed to retrieve articles related to the disease of interest, just as we did in our methodology. For NER, GeneWizard recognizes the biological entities related to a disease by using dictionaries created for the disease and for the genes. To identify relationships between genes, GeneWizard performs clustering of the abstracts, based on similarity matrices constructed from abstracts, based on the frequencies of the disease and gene terms.

Another goal of GeneWizard is to be highly usable, so that not much experience with text mining methods is required of the users. Faro et al. [11] stress that it is important for tools that generate biological hypotheses to have a high usability, since the audience who use these tools are likely to be biologists, not computer scientists.

Another tool is called BioWizard, which is very similar to GeneWizard, yet it performs full-text analysis instead of abstract analysis [23]. Also, BioWizard was tested against gold standard gene-disease relationships in order to check the precision of the recall, in addition to experimental data in the form of microarray data. This system was then moved to the cloud in order to perform more intensive computations in a shorter amount of time [24].

Another study which generated hypotheses from literature performed the IE step by splitting the abstracts into sentences and considered the sentences which contained an interaction plus two gene names [25]. A network of genes was built from the extracted genes and interactions. The genes which ranked the highest in centrality measures were manually validated by looking through literature. A similar study was done by [6], and high accuracy was achieved for finding actual gene-disease relationships in prostate cancer. Interestingly, even genes which were missed later turned out to have an article written about how they were indeed involved in prostate cancer [6].

Our contribution is that we will use different data mining techniques and various APIs for the different stages of the text mining, and that we will investigate relationships such as gene-country, gene-year, and abstract-country which have not been investigated by other papers so far. We explored how these new types of relationships can help to generate hypotheses about which genes should be studied.

## Methods

### The developed solution

#### Overview

**Fig. 3:**
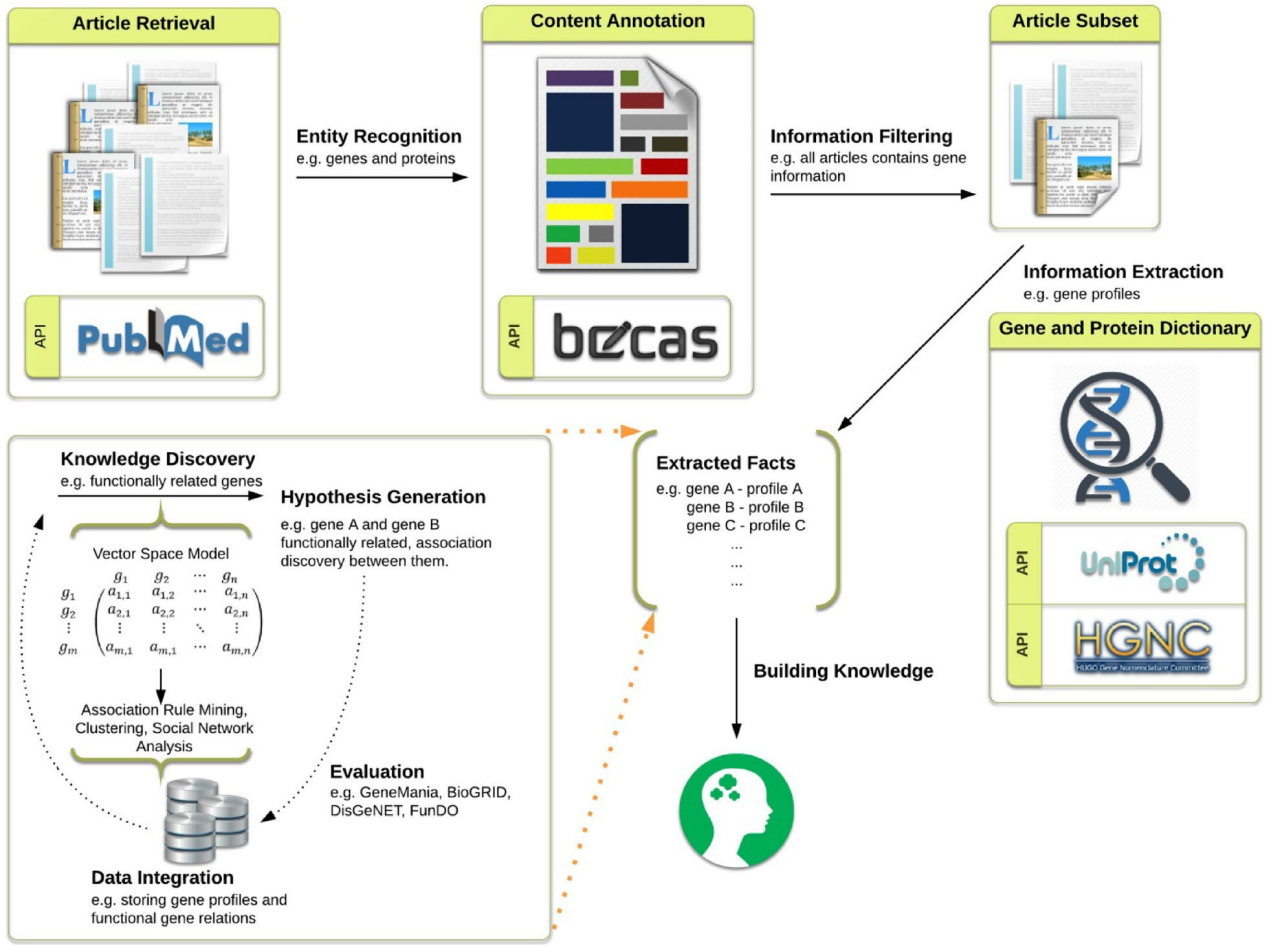
Outline of the workflow and resources used in the proposed solution

Figure 3 illustrates the steps of the methodology. Our goal is to contribute novel ideas for KD and hypothesis generation related to genes involved in breast cancer. We decided to use ready-API’s for IR, NER, and IE parts of the developed framework. The first step in our solution was the IR step, where our goal was to retrieve all relevant papers related to our topic of interest: breast cancer.

Although full-text analysis contains more information than abstracts [11, 12, 14], we chose to examine abstracts because they contain the most important and concise keywords. Also, due to their shorter length, their analysis would be much faster to compute, so this would enable us to do a larger scale text analysis. Moreover, we speculated that full-texts may contain references to other genes which are not necessarily related to breast cancer, or genes that may be relevant to other cancer, which may add to the noise. In other words, although full-text mining may produce a higher recall, abstract based text mining may produce a higher precision. Therefore, our first step was to retrieve as many biomedical abstracts related to breast cancer as possible. All of the abstracts which we used for the analysis were retrieved using the PubMed API to the MedLine database. We chose to use PubMed because it is the most well-known search engine for biomedical papers [11, 12, 14]. The search keywords that we used were “breast cancer”. The total amount of abstracts which were retrieved from PubMed was 289,510 in the month of October, 2014. We then filtered the papers so that the remaining subset of 225,059 that had an abstract, title, authors, and a journal name. Of the paper set that was excluded, 62,752 papers did not have an abstract and 257 did not have a date.

The PubMed API also provided extra information about the articles, such as keywords, title, abstract, authors, affiliation of authors, publishing date, and journal name. In addition to the abstracts, it was useful to receive most of the extraneous data in a standardized format, because we could use it to perform additional analysis on breast cancer data. However, not all of the data was clean and therefore they required more processing, such as author affiliation. We will later discuss how we processed author affiliation in order to use it for the analysis. In the next step, we recognized the named entities in the abstracts and titles. We used an online API called BeCAS, which identifies biomedical concepts in text [16]. In our opinion, BeCAS is a well-documented API; it performs well enough at identifying biomedical terms. Further, another important reason for using BeCAS was because it is integrated with PubMed such that it requires only the PubMed ID of the abstract in order to perform the analysis. Thus, we did not need to upload the abstract itself into BeCAS. This saved computational memory and time.

The named entities we were interested in are genes and proteins. Since we wanted to consider only genes for our analysis, we collected the genes from the text, but we also collected genes which were associated with proteins that were mentioned in the text. Another reason for using BeCAS is because it is well-integrated with the UniProt database [26] which stores genes and proteins information. For each protein and gene, BeCAS provided the UniProt ID in order to verify the entity. The UniProt ID also allowed us to retrieve genes which were associated with the proteins mentioned in the text. UniProt also helps to address one of the biggest challenges in biomedical text mining, i.e., genes may contain many synonyms. UniProt stores known synonyms for each gene name. This helps to reduce the number of duplicate genes listed within the abstracts under alternative names. After recognizing genes within the abstracts as well as those associated with the proteins mentioned in the abstracts, we filtered the paper set to include only abstracts which contain genes. Therefore, our final paper-set used in the analysis was reduced to 117,339 papers. The abstracts which were excluded following the NER step may be related to other aspects of breast cancer, possibly from a health care or psychological perspective, not the genetic side which we are interested in.

The next step was to generate hypotheses about the relationships between genes, and also between genes and other information associated with them, such as the abstracts and authors. The relationships between the genes were measured as co-occurrences within the abstracts, and the semantic relations or directionality between the genes were not extracted to be used in the analysis. Although many hypothesis-generating methodologies use gene–gene relationships to generate hypotheses about which genes should be investigated, our methodology uses additional information, such as the authors, locations, and dates. Therefore, we developed a methodology to create hypotheses that stem from different types of information that is typically used by other researchers.

One of the features that we examined was the country of an author’s affiliation. By extracting the country of an author’s affiliation, we then related the countries which published breast cancer papers to the genes. Interesting correlations were then found, such as the genes that particular countries focused on. Researchers might use the gene-country information to see which genes are hot topics to study in a country. Another feature that we considered was the year that the abstract was published in. The gene-year relationship allowed us to find which genes were frequently mentioned together every year, which might lead a researcher to believe that these genes might have a hidden connection that needs to be further explored in the wet-lab. A third relationship that we explored was gene–gene co-occurrence frequency within the abstracts. An ideal analysis technique to explore the gene–gene relationships was network analysis, as the genes could be the “actors” and the number of abstract co-occurences could be the “action” between two genes. The network analysis technique is further discussed in “Results and discussion” section. Lastly, we also examined how many abstracts each country published in order to find which countries are the top contributors to breast cancer research.

For the data mining analysis, we used the software KNIME.^1^ For the social network analysis, we used Gephi.^2^ The web tools that we used to evaluate some of our results were GeneMania, DisGeNET, and FunDO. The computer used for the analysis has the following main specifications: Intel i5-4570 CPU, 8gb RAM, Windows 10 OS.

#### Country identification

**Fig. 4:**
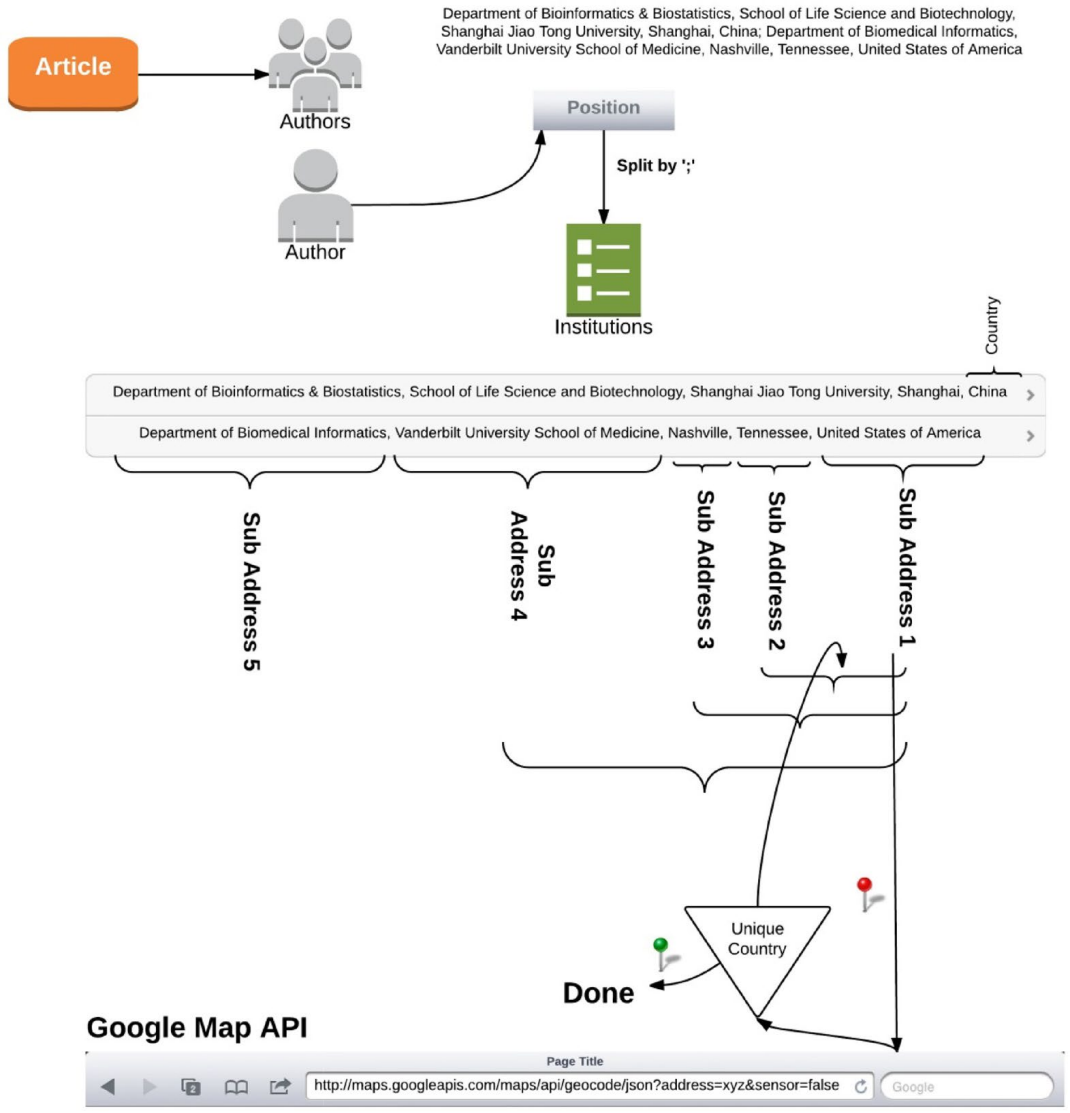
The extraction of the country

To find countries associated with each retrieved article, we needed to process the string which contains the affiliation(s) of authors, called the position (Fig. 4). The extra processing was required because the position often contained extraneous information, such as the names of the institution(s) and the author’s e-mail. The number of authors was around 500,000, but after we grouped them by first name, last name, and affiliation, the number rose to 601,287, most likely due to authors changing institutions throughout their careers or having popular names referring to different authors at different institutions, e.g., ‘Ken Barker’ is a popular name who exists at three institutions. There were 193,000 different possible affiliations for the authors who published abstracts with genes mentioned in them. Many authors contained multiple institutions in their affiliations.

For each of the affiliations, we then wanted to find the associated country name. We used google maps API^3^ to retrieve the country name. We split the string into sub addresses using the comma delimiter. Each search was performed using the rightmost delimited address, which often contained the country name. However, when the sub address string was insufficient to achieve exactly one country name, we repeatedly increased the size of the string with the next rightmost element of the sub address. As seen in Fig. 4, we first made a query using sub address 1, and if that did not return precise enough results to reveal the country of origin, then we made another query which also included sub address 2, etc. The final set excluded all of the institutions inside each affiliation which did not contain a valid address, which was about 1 %. One limitation of google maps API is that it had a daily quota of queries which could be submitted to the service. With our large number of institutions, we needed to optimize the number of online queries. We achieved this by constructing a cache system which stored all special keywords existing in the affiliations; this helped us to distinguish the institutions directly. Using the cache system, we submitted only 8558 queries to google maps API. Altogether, we found that there were 159 countries with articles published under “breast cancer” category and contain genes.

## Results

### Evaluation of the developed solution

#### Overview

Our gene–gene results were evaluated by comparison to results retrieved through a web tool called GeneMania^4^ which uses publicly available curated and experimental data to derive gene–gene relationships [19]. GeneMania also shows predicted relationships [19]. If most of the relationships that we hypothesize are also reported by GeneMania, then our hypothesis would be strengthened. Any gene–gene relationships that are missing in the GeneMania results have the potential to be newly discovered relationships that may warrant more investigation by wet-lab researchers.

Our gene-disease results were evaluated by comparing our results to DisGeNet and FunDo, which are two web tools that identify gene-disease relationships.

#### Resources used

##### Evaluation of gene clusters and communities

For the evaluation of our results, we used GeneMania in order to link our text-mining results to results drawn from experimental data [19]. GeneMania accounts for a few different types of interactions between genes, such as co-expression, physical interaction, genetic interaction, shared protein domains, co-localization, pathway, as well as predicted relationships using orthological functional data from other organisms. For all of our evaluations, we used datasets that described human genes.

Co-expressed genes are genes which had the same expression levels over the same conditions in a published study, where most of the gene expression data came from the gene expression omnibus (GEO) database. Another interaction in GeneMania is physical interaction, which means if two genes code for proteins that have a physical interaction, then the two genes have a connection. These protein–protein interactions were pulled from BioGRID^5^ and pathwaycommons databases, which store protein–protein interactions. The other interactions we considered from GeneMania were shared protein domains, Co-localization, and pathway interactions. Two genes partake in the shared protein domain interaction if their proteins have the same protein domain. Two genes have co-localization interaction if their proteins are found in the same body tissue. Finally, two genes share in the pathway interaction if they participate in the same reaction in a pathway. The sources of data that GeneMania uses are listed in the highly cited published paper [19].

##### Disease identification

To find the disease which was most associated to each gene, we used the DisGeNET^6^ API [20, 21]. DisGeNet finds gene-disease relationships, from either curated sources, literature based associations, or predicted associations. For our study, we were interested only in human gene-disease relationships, so therefore we only used the curated sources. The curated sources for DisGeNET include human gene-disease relationships from the comparitive toxigenomics database (CTD) and UniProt. We used DisGeNET to find the gene-disease associations for the genes found through the gene-year and gene-country clustering (Appendix: Tables 9, 10). The diseases were identified on a gene by gene basis.

For the social network analysis, we used FunDO^7^ to identify the diseases which were common between large groups of genes [22]. FunDO takes a list of genes and retrieves the related diseases, based on the disease ontology database. The reason that we used FunDO instead of DisGeNET for analyzing the gene communities, is that FunDO provides a better analysis for common diseases between a group of genes. DisGeNET provides exclusive lists of diseases for each gene, whereas FunDO provides a list of shared diseases among the genes. An automated identification of diseases shared among groups of genes was beneficial, because the smallest community we obtained had 229 genes in community 1 (Appendix: Table 8). For each community from the social network analysis, we retrieved the top five diseases within the community.

## Discussion

### Results and discussion

#### Hard clustering

Clustering is the process of grouping items together into “clusters”, so that the items within each cluster have more similarity to each other than to items in other clusters. Hard clustering separates items into distinct groups, where each item belongs to exactly one cluster. We performed hard clustering on genes with respect to the country affiliation of the authors who published papers on the genes. In this section, we present our results and some of the interesting genes a researcher might find to study from the results.

##### Which countries have studied the largest number of breast cancer genes?

**Fig. 5:**
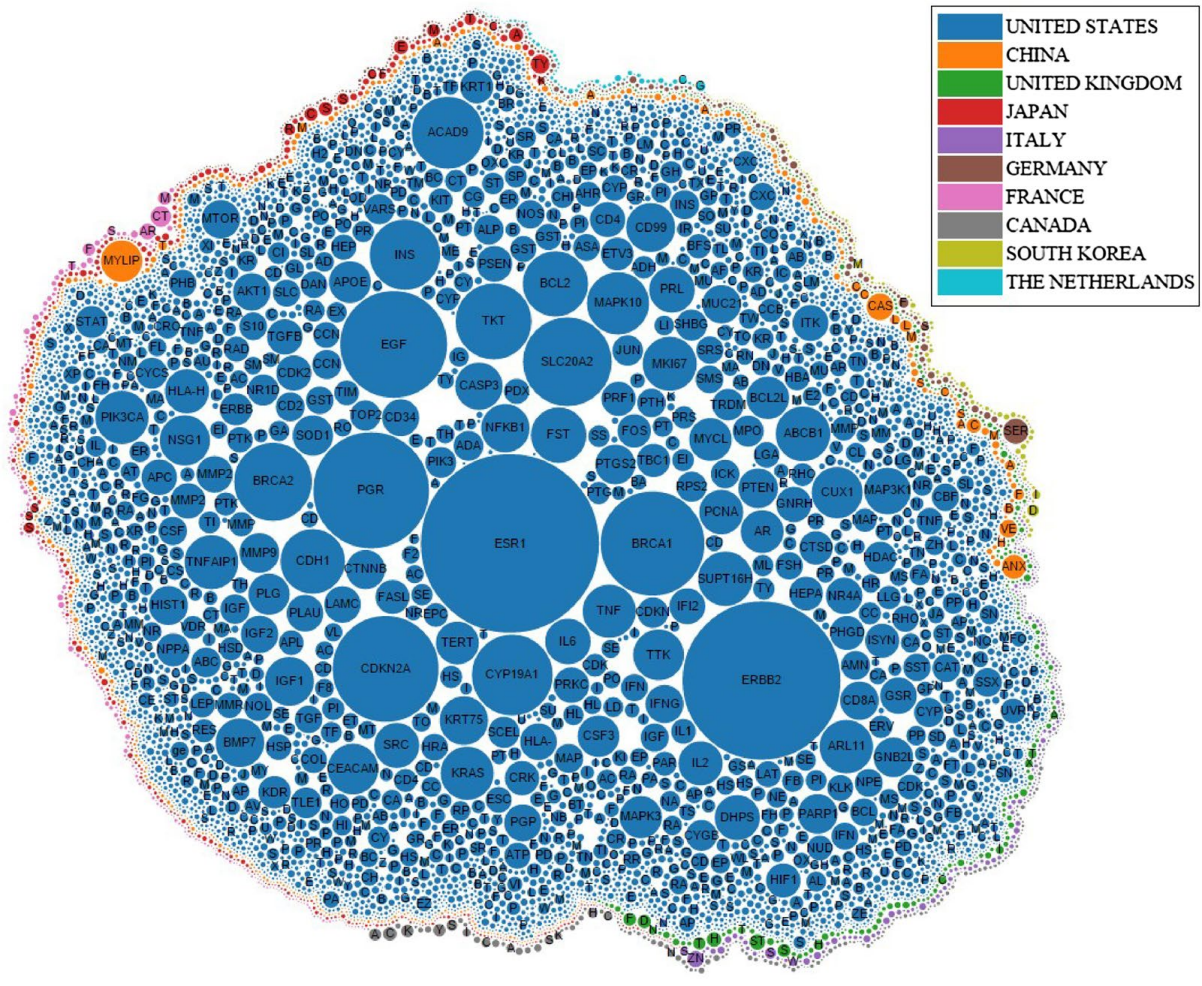
The top 500 most frequently mentioned genes are shown, where *radius* represents the number of abstracts which mentioned the gene, and the *colour* represents the country which mentioned the gene the most

In Table 1, the country which published the largest number of articles on the topic of breast cancer is the United States; authors affiliated with the United States also published the largest number of articles which mention breast cancer genes. In Fig. 5, the genes were clustered by colour of the countries that published the most amount of papers on those genes. Figure 5 shows that the United States has studied the largest number of genes by far, since most of genes have been mentioned by abstracts affiliated with the United States. Countries which ranked second and third are China and United Kingdom respectively. The United States, United Kingdom, and China seem to have the largest support for breast cancer research and are leading the research worldwide.

**Table 1:** The number of gene mentions

All Abstracts		Abstracts with gene mentions	
United States	62,013	United States	33,373
United Kingdom	11652	China	6553
China	8858	United Kingdom	6041
Japan	8807	Japan	5299
Italy	8667	Italy	4621
Germany	7394	Germany	4148
France	6757	France	3642
Canada	6476	Canada	3573
The Netherlands	4071	South Korea	2144
Australia	3601	The Netherlands	1844

**Table 2:** Represented 5 highest maximal closed frequent item sets for Gene-Country

Gene maximal closed frequent item set	Support
ERBB2, ESR1, PGR	48.43
EGF, ERBB2, ESR1	46.54
BRCA1, ERBB2, ESR1	45.91
BRCA1, BRCA2	45.28
CDKN2A, ESR1	45.28

**Table 3:** Represented 5 highest maximal closed frequent item sets for Gene-Year

Gene maximal closed frequent item set	Support
CEACAM3, ESR1	82.69
ALPPL2, CD99, CEACAM3, CHI3L1, ESR1, MUC21, SOD1	78.85
AMN, CD40LG, CD79A, CEACAM3, ESR1, PRL	78.85
AFP, CEACAM3, ESR1	76.92
CD99, DHPS, POMC	76.92

**Table 4:** Top 10 diseases associated with genes derived from the union of the top 5 gene-year and gene-country itemsets

Disease name	Genes
Breast neoplasms	ERBB2, ESR1, PGR, EGF, BRCA1, BRCA2, CD99, AFP
Adenocarcinoma	ERBB2, PGR, EGF, CDKN2A, CD99
Mammary neoplasms, experimental	ERBB2, PGR, BRCA1, AFP
Carcinoma	ESR1, PGR, BRCA1, CD99
Prostatic neoplasms	ERBB2, EGF, BRCA1, BRCA2
malignant neoplasm breast	PGR, BRCA1, BRCA2
Glioma	ERBB2, CDKN2A, CHI3L1
Hypertension	CHI3L1, SOD1, POMC
Neoplasm	BRCA1, CDKN2A, CD99
Ovarian neoplasms	ERBB2, BRCA1, BRCA2

**Table 5:** Statistical information for gene–gene network

	Nodes	%	Edges	%
Full network	8400	100	213,894	100
Giant component	7620	90.71	213,877	99.99
Pruned giant component	1089	12.96	6815	3.19

**Table 6:** Network Analysis measurements for the gene–gene network

	Betweenness centrality	Modularity class		Closeness centrality	Modularity class
ESR1	0.09	2	ESR1	0.62	2
ERBB2	0.06	2	ERBB2	0.6	2
CDKN2A	0.04	6	CDKN2A	0.58	6
SLC20A2	0.03	2	SLC20A2	0.57	2
EGF	0.02	2	EGF	0.57	2
PGR	0.02	2	PGR	0.56	2
BRCA1	0.02	6	ACAD9	0.55	5
CDH1	0.02	0	CDH1	0.55	0
ACAD9	0.02	5	MAPK10	0.55	5
HLA-H	0.02	6	TKT	0.55	2

The top 10 genes with the highest betweenness are shown, as well as the top 10 genes with the highest closeness. The modularity class is also shown, where it denotes the community that the gene belongs to

**Table 7:** Common diseases in each community

	Cancer	Breast cancer	Prostate cancer	Diabetes mellitus	Colon cancer	Obesity	Leukemia	Hypertension	Atherosclerosis	Rheumatoid arthritis	Embryoma
Community 0	X	X	X	X	X						
Community 1	X	X	X	X		X					
Community 2	X	X	X	X			X				
Community 3	X	X	X	X	X						
Community 4	X	X		X				X	X		
Community 5	X	X	X	X						X	
Community 6	X	X	X		X						X
Community 7	X		X	X			X			X

In general, the difference between the top countries which published articles pertaining to breast cancer was not very different from the top countries which published articles containing breast cancer genes. Therefore, in these top countries, the molecular side of breast cancer was just as studied as are other aspects of breast cancer; this shows the importance of genetics in breast cancer research.

##### Collaborations

**Fig. 6:**
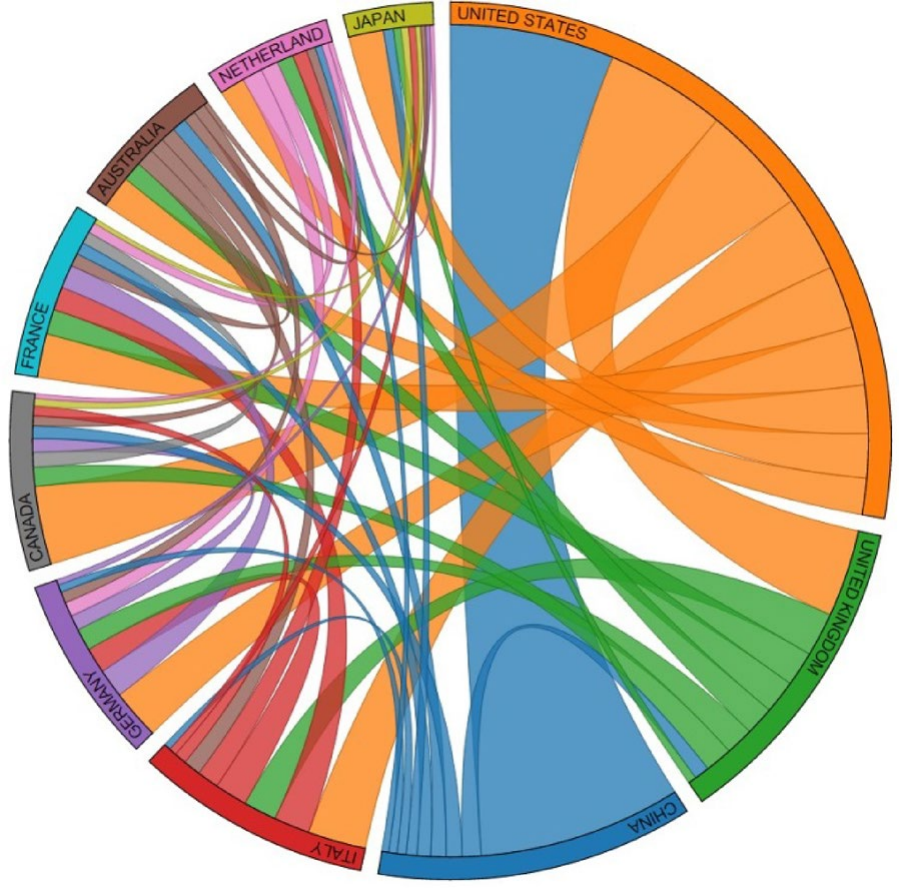
Collaboration between the top 10 countries in regard to breast cancer abstracts

**Fig. 7:**
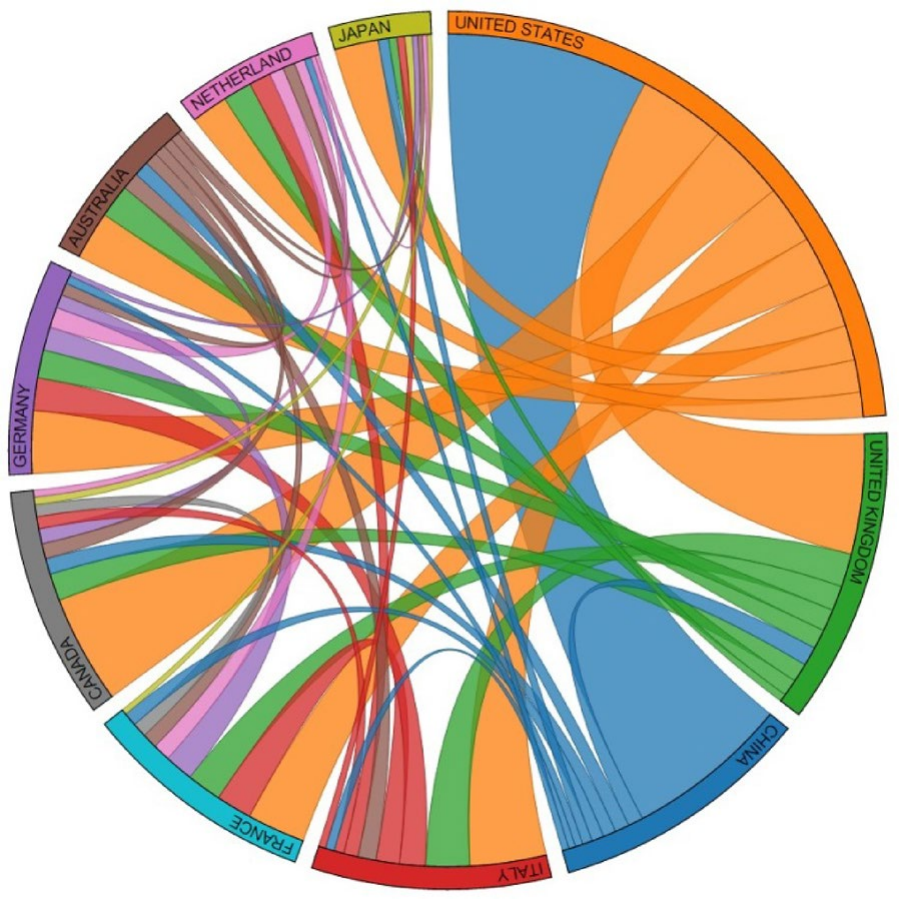
Collaboration between the top 10 countries in regard to breast cancer abstracts that contain genes

We assume a collaboration if a paper had affiliations with institutions in different countries. The number of collaborations between countries on articles which had to do with breast cancer occurred most likely between United States and China (see Fig. 6). However, when we considered collaborations on articles which mentioned breast cancer genes, countries which had the largest number of published articles such as United States, United Kingdom, and China had a slightly lower number of collaborations. However, countries with a lower amount of publications had more collaborations than before (see Fig. 7). Collaboration information allows researchers to recognize countries which are most involved in research as a partnership with others.

##### What are the top studied genes in the breast cancer field?

**Fig. 8:**
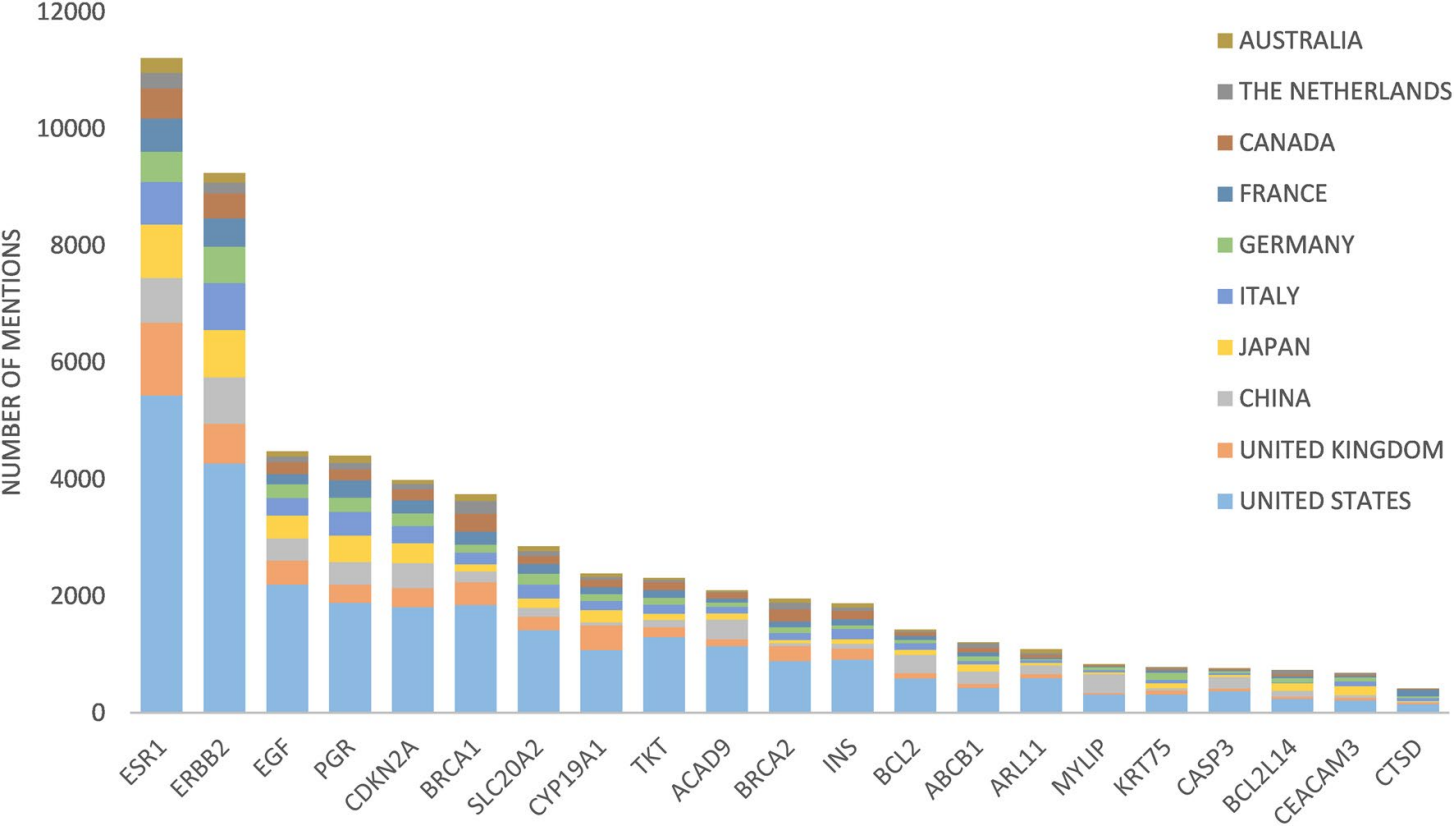
For the top 21 most frequently mentioned genes, the distribution of gene mentions by country is *colored*

Researchers may want to know the top studied genes in the breast cancer field, so that they may focus their research on promising genes. The top two most mentioned genes in the breast cancer abstracts were ESR1 and ERBB2 (Fig. 8). The next five most studied genes were EGF, PGR, CDKN2A, BRCA1, and SLC20A2 (Fig. 8). In total, there were 21 unique genes, when we considered the top 10 most studied genes for the top 10 countries in breast cancer research. Related to these genes, more detailed information is listed in Appendix: Table 11. However, please note that the curated source from DisGeNET did not contain information for CEAMC3, MUC21, and DHPS.

**Fig. 9:**
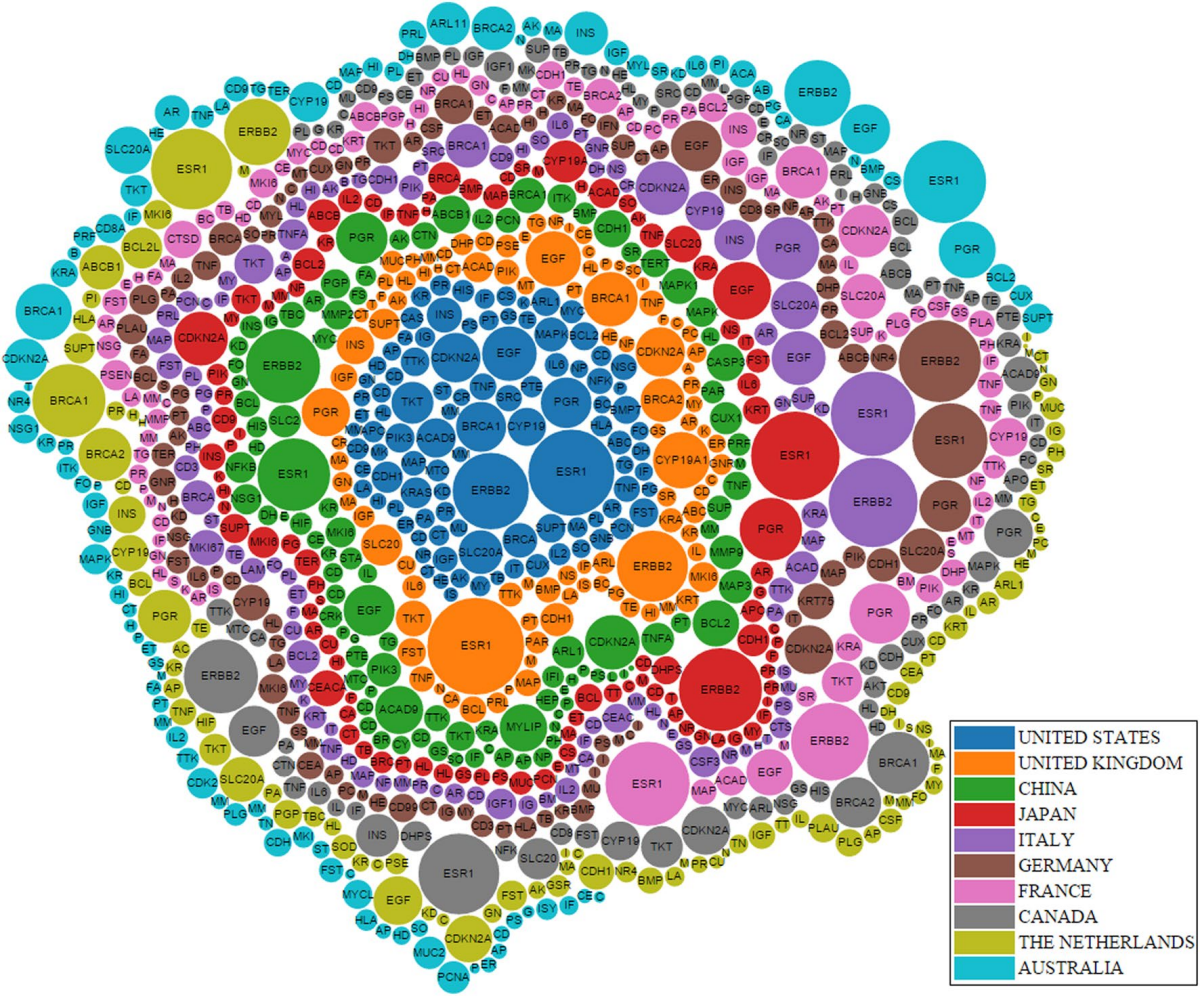
The division of effort by the top 10 countries, for the top 100 genes for those countries

To measure the amount of effort that a country X put into a gene Y, we divided the number of abstracts from country X which mentioned gene Y, by the number of papers published from country X. All of top 10 countries for breast cancer research put most of their effort into ESR1 and ERBB2 (Fig. 9). Gene ESR1 received 11–20 % of the effort, with the United Kingdom contributing the highest effort. Gene ERBB2 is contained in 9–17 % of the effort, with France contributing the highest effort. For all the 21 unique genes, the effort ranged from 2–20 %.

Unsurprisingly, the protein products of ERBB2 and ESR1 are targets of drug and hormone therapy for breast cancer.

ERBB2, popularly known as HER2, codes for a receptor tyrosine-protein kinase, which is found in membrane signaling complexes, and facilitates the transmission of cell messages [27]. If ERBB2 is over-expressed, then the cell may get too many messages to proliferate and to survive, which may lead to breast cancer. Breast cancer patients which are ERBB2 positive (30 % of patients) can be treated with the medication trastuzumab, with the trade name Herceptin [28].

On the other hand, ESR1 codes for the first out two types of estrogen receptors, which is found in breast cancer cells.

The estrogen receptor is a transcription factor found in the cytosol, but when activated by the hormone estrogen, it can move into the nucleus and regulate growth and proliferation genes. Estrogen receptors are over-expressed in about 70 % of breast cancer cases. [29]. Three hormone drugs that are used to block estrogen receptors are tamoxifen, toremifene (fareston), and fulvestrant (faslodex) [29, 30].

We were also interested to find whether some countries had a greater interest in some of the genes, as compared to other countries. For this analysis, we wanted to avoid genes that had been sparsely studied, so that the results would not be skewed. For example, consider the situation where gene X has only been mentioned in two abstracts and studied by two countries. Then the results would indicate that one of the countries has invested much effort into this gene, although that country may have only published one paper on the gene. Therefore, we analyzed the top 21 genes, where the number of abstracts for each gene ranged from 419 to 11,215.

**Fig. 10:**
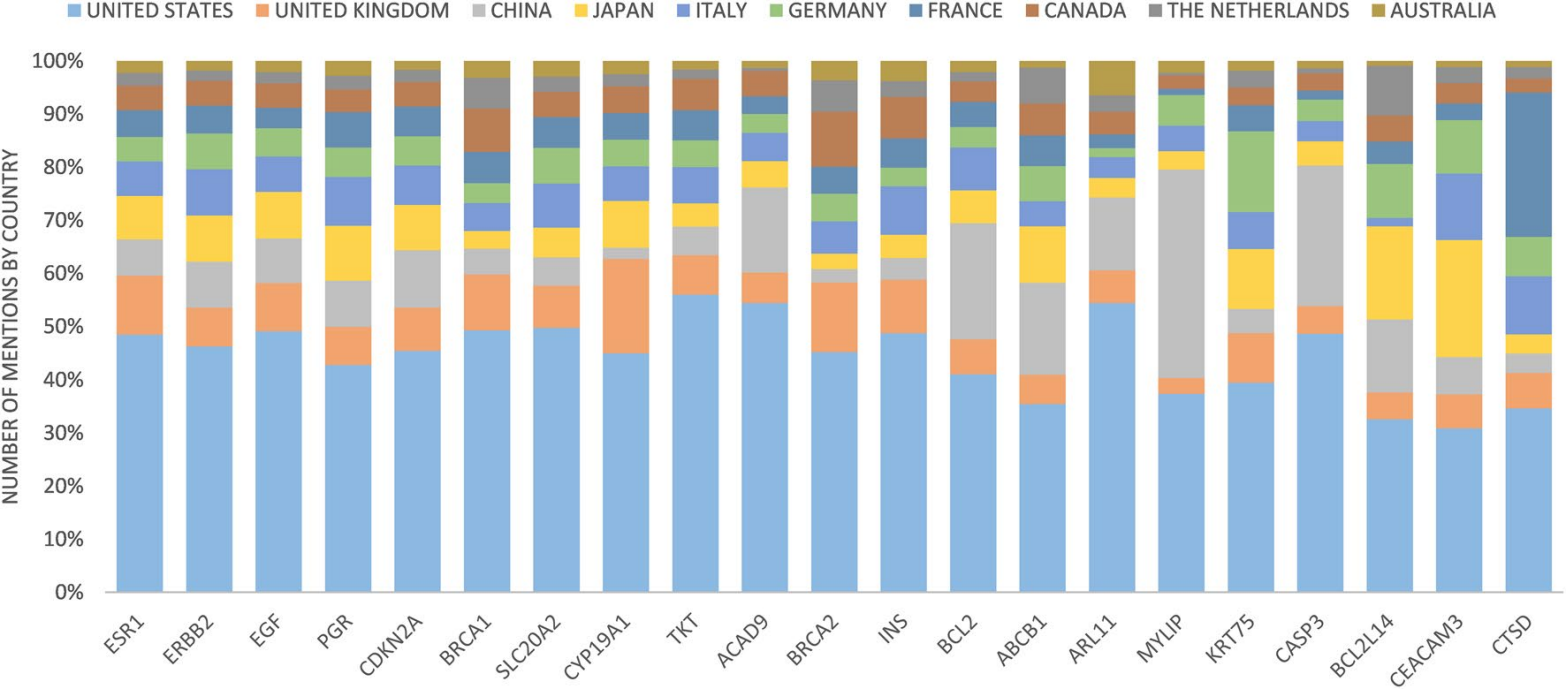
The proportion of gene mentions by each country

When considering the number of abstracts, the United States has published the greatest number of papers for each gene, except in one case (Fig. 10). For gene MYLIP, China has more abstracts than United States, with 327 versus 312. Notably, there are some countries that follow closely behind the United States for some of the genes. For gene CEACAM3, the United States has 212 abstracts and Japan has 151. For gene CTSD, the United States has 145 abstracts, and France has 114.

**Fig. 11:**
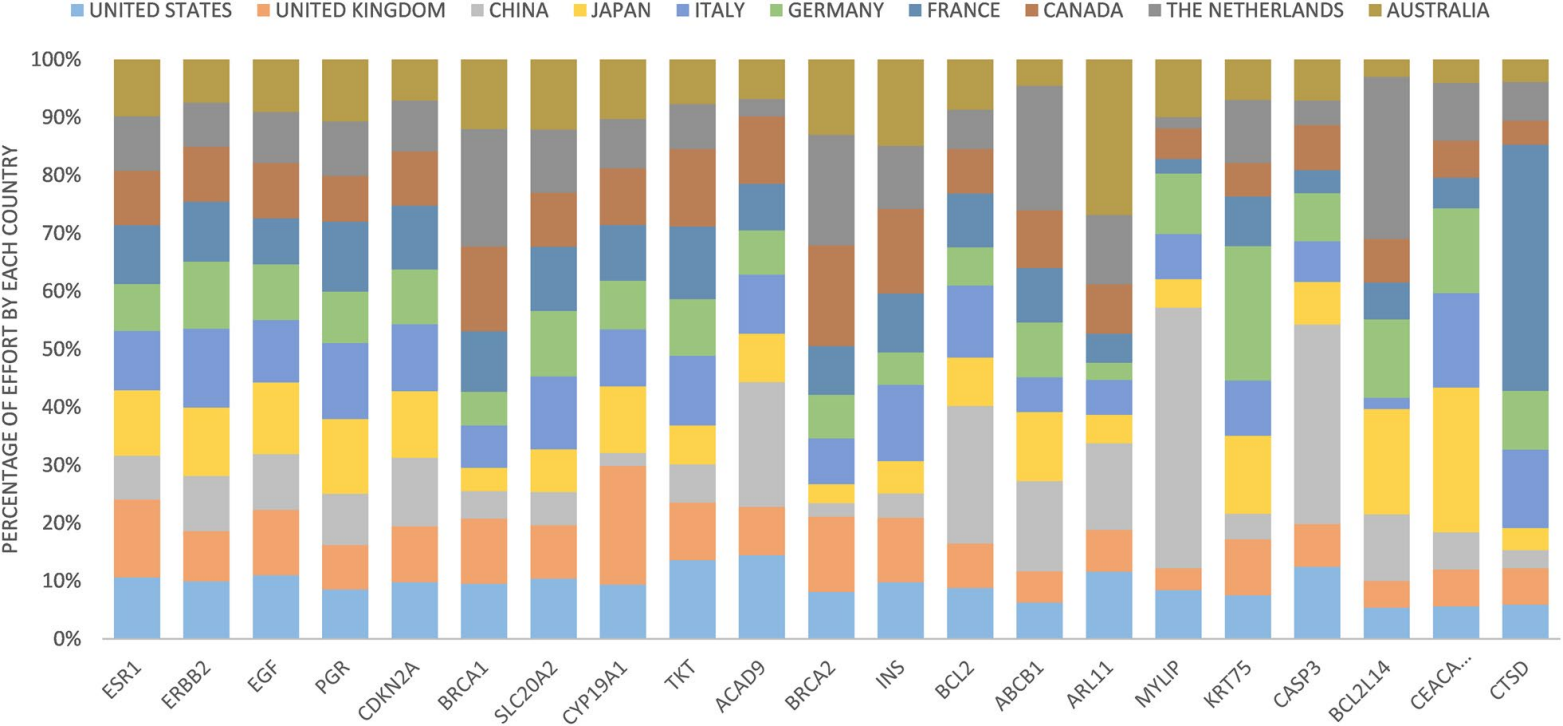
In consideration of other genes that these countries have studied, this figure shows how much of that effort was placed on these genes

However, when considering the effort put into each gene, the United States did not hold the largest proportion of effort (Fig. 11). Since the United States has published a lot of work on many genes, then the amount of effort for each gene decreases. For example, although the United States has published five times more papers than the United Kingdom on gene ESR1, the United Kingdom placed 20 % of its effort into gene ESR1, whereas the United States placed only 16 %. Information on country effort can be useful to find the priorities that each country places on the genes, relative to other countries.

The *MYLIP* gene has seen more priority from China, with 5.0 % of China’s research effort into these gene, versus 0.2–1.2 % of effort coming from other countries (Fig. 11). MYLIP also had more papers overall coming from China, rather than the United States, so this gene seems to be quite important for Chinese affiliated research. Although MYLIP does not appear to be a drug target, it seems to be upregulated by tamoxifen [31].

MYLIP codes for a myosin regulatory light chain (MRLC) interacting protein [32]. The MYLIP protein mediates ubiquitination, which is followed by degradation of the MRLC. When the MRLC is degraded, then neurite (an axon or dendrite of a neuron) outgrowth is also inhibited.

Some other genes that received more interest and priority from particular countries were ARL11 and 4.1 % of effort from Australia, CASP3 and 3.1 % of effort from China, BCL2L14 and 3.7 % of effort from The Netherlands, CEACAM3 and 2.8 % of effort from Japan, and CTSD and 3.1 % effort from Italy (Fig. 11).

An interesting point to consider is how regulated breast cancer research is in each country. If the direction of breast cancer research is tightly regulated in some countries, then our study of publication effort towards the genes may reveal that direction. One way that the government of a country might regulate breast cancer research is to encourage funding for groups which are studying particular genes. Promising genes to study might be the ones which have high potential for target drugs, or the ones that have a higher impact on breast cancer for that country’s population.

One limitation is that that our paper set may also include genes which have only been studied in mouse or rat models. Therefore, it may be difficult to confirm how these genes have a relationship to breast cancer in humans.

##### Which genes were never mentioned by the top 10 countries?

In total, there are 445 genes which were not mentioned in any of the abstracts written by the top 10 countries. The largest frequency of a gene not mentioned in the abstract of a top country is seven abstracts. Such a low frequency of seven, as compared to 18,913 for the *ESR1* gene, indicates that the top 10 countries covered most genes. However, examining these genes may be interesting to to understand whether they have the possibility to be candidate genes or if they are outliers. To test this, we closely inspected some of genes, such as GLCE, which has abstract frequency of seven.

Gene GLCE codes for a protein called d-glucuronyl C5-epimerase, an enzyme which biosynthesizes the carbohydrate portion of heparan sulphate proteoglycans (HSPGs) present on cell surface [33]. Enzymes which biosynthesize cell-surface sugar have the potential to be implicated in cancer growth because cell-surface sugar and proteins (proteoglycans) are involved in signalling to cells. Signalling may indicate to a cell whether it should divide or not. If genes or proteins which have a role in such a signalling pathway are defected, then the cell may begin to divide infinitely, and therefore become cancerous.

Interestingly, in one of the few research articles that mentioned GLCE, it was shown to have an antiproliferative effect on breast cancer cells. It was found that the down-regulation of GLCE may indeed lead to breast cancer [33]. Therefore, the case study of GLCE shows that although some genes may not be mentioned as frequently as others in the abstracts, they still have potential to be important genes to breast cancer.

Another example is *CHRM1* gene, which had a frequency of five abstracts. However, CHRM1 seems to be much involved in prostate cancer [34]. It codes for an acetylcholine receptor involved in the autonomous nervous system. Again, cell-surface receptors have a high potential to be involved in cancer because they form a crucial part of cell signalling. CHRM1 has been shown to have an effect on prostate cancer in a high-impact article with 56 citations to date, although it was published in 2013 [34]. Therefore, another reason that some genes may have a low mentioning in the abstracts is that they have been shown to be important in another cancer, yet researchers are only recently investigating their connection to breast cancer. Genes which are not mentioned in many breast cancer abstracts may guide researchers to genes which require further investigation. With more research invested in these other genes, they may prove to be important biomarkers for breast cancer.

#### Hierarchical clustering

Hierarchical clustering is used to build a hierarchy of clusters, where two possible similarity measures that can be used are single-link and complete-link [8]. From a high-level perspective, Single-link clustering produces clusters based on how similar the items are to one another, whereas complete-link clustering produces clusters based on how dissimilar the items are.

**Fig. 12:**
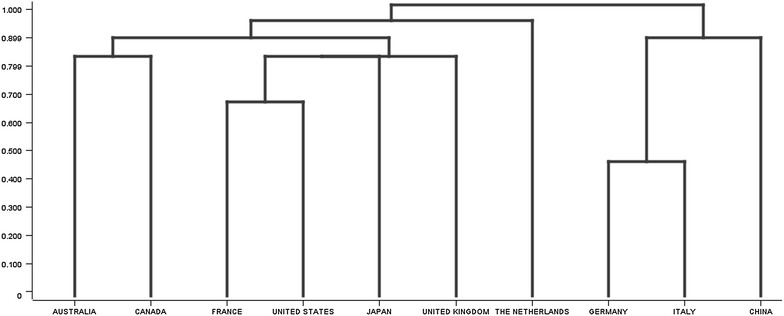
Hierarchical clustering of the countries, based on the genes that each country studied. This figure shows how similar the research interests are across the countries

We applied hierarchical clustering between the countries, based on the genes that each country studied. We used the complete-linkage measure, because this measure has the advantage or producing more compact clusters, which leads to a clearer hierarchy. Our clusters were already very similar to each other, so we wanted to create more separateness. The results of the hierarchical clustering are displayed in Fig. 12. The hierarchical clustering revealed that Germany, Italy, and China formed one branch, and then the second branch was formed United Kingdom, Japan, United States, France, Australia, and Canada. Lastly, a third branch was formed by the Netherlands. A researcher can use Fig. 12 to see which countries have research interests in common.

#### Frequent pattern mining

Frequent pattern mining is used to find sets of items that occur frequently together in a database, and is often applied in grocery stores to discover which items the customers tend to purchase together [8]. Different algorithms such as apriori and FP-growth may be applied to generate frequent item sets from a collection of transactions. We applied the FP-growth algorithm to find the frequent item sets using the tool KNIME.

One measure of significance for item sets is support. Support is a decimal value that represents the proportion of transactions in the database that contain a particular item set. For example, if the item set A, B, C is found in 10 % of all transactions, then that item set has a support of 0.1.

To produce more concise and pruned results, we additionally considered other constraints on the item sets, where each of the item sets had to be maximal closed. An item set is maximal if none of its super sets are frequent, and an item set is closed if none of its super sets have an equal support value. For an additional explanation of maximal closed item sets, please refer to [8].

##### Genes frequently mentioned together by countries

**Fig. 13:**
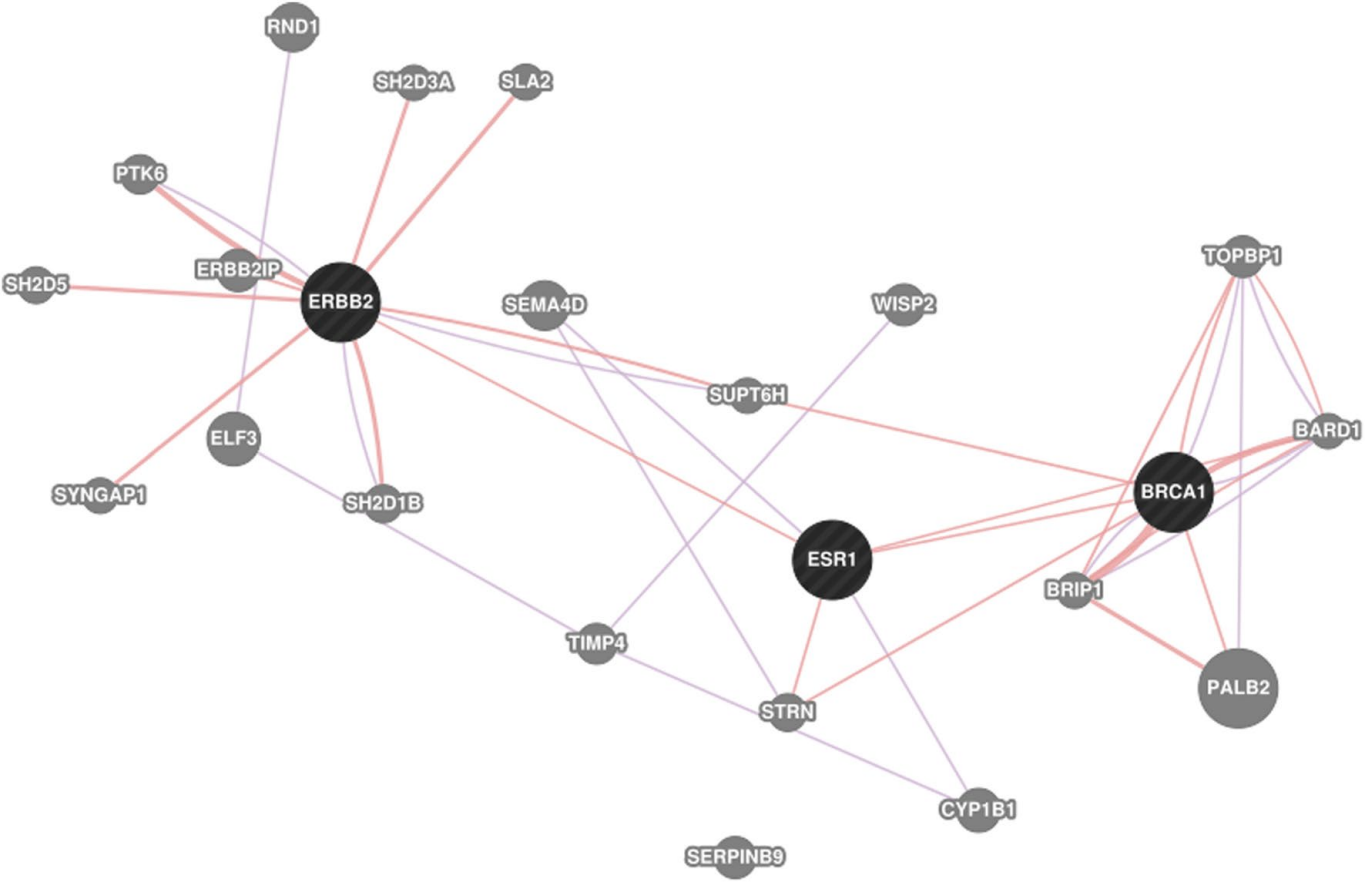
*Black nodes* are genes listed in the third gene-country item set in Table 2. As described by GeneMania, the *purple connections* represent co-expression, whereas the *red connections* represent physical interaction between the gene products

We computed the maximal closed frequent item set to find which genes are frequently mentioned together by each country. We arbitrarily considered the top five item sets and they are listed in Table 2. We then took a closer look at the item set which contained the following genes: *BRCA1*, *ERBB2*, *ESR1*. In Fig. 13, we used GeneMania to show that there is a relationship between the aforementioned genes, as found in the gene expression data and the literature. Red edges represent physical interaction, and purple edges represent co-expression.

##### Genes frequently mentioned together every year

**Fig. 14:**
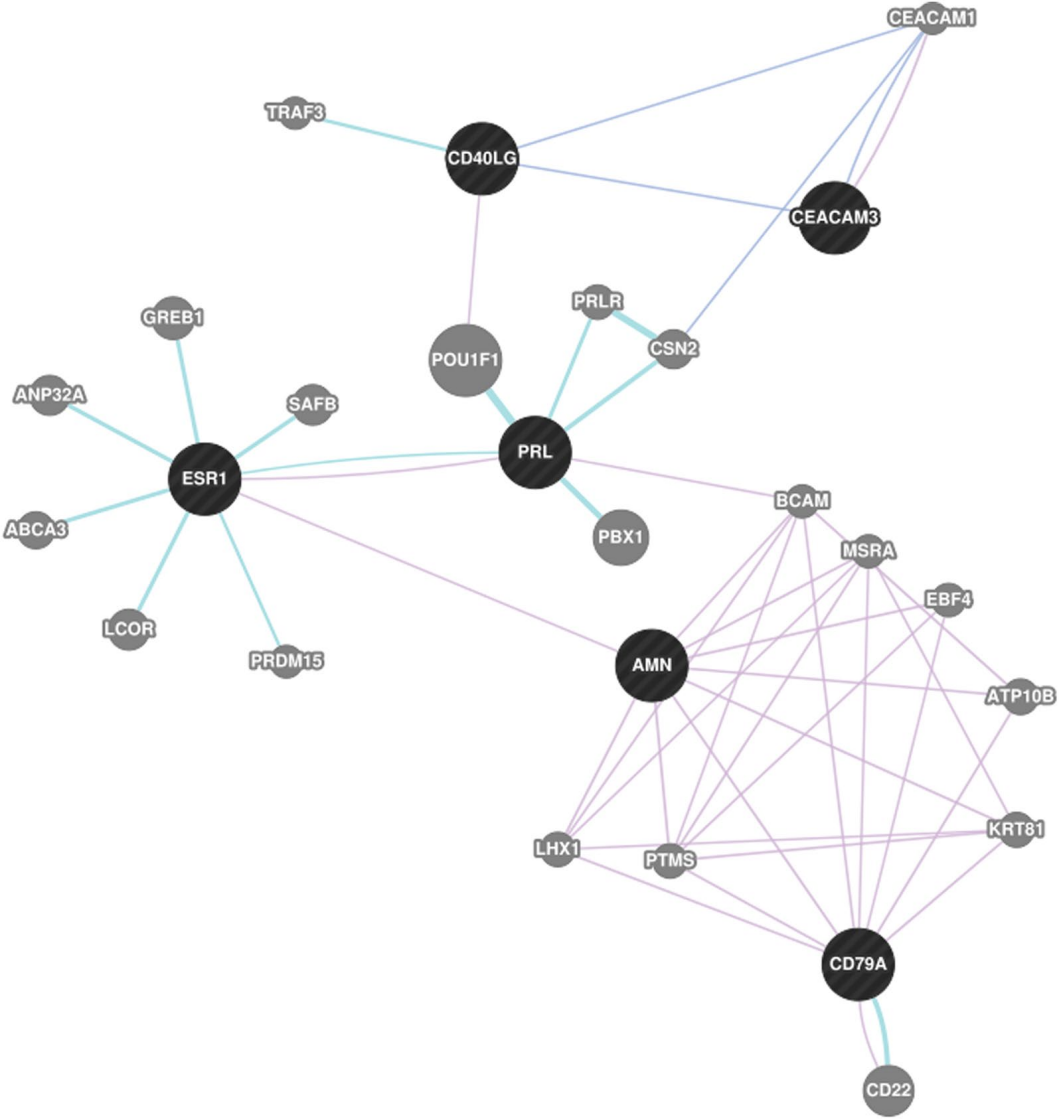
*Black nodes* are genes listed in the third Gene-Year item set in Table 3. The connections between the genes are described by GeneMania as *blue* for co-localization of the gene products and *purple* for co-expression of the genes

Again, we computed the maximal closed frequent items sets for genes that are mentioned together every year. We arbitrarily considered the top five item sets and they are listed in Table 3. We then took a closer look at the item set which contained the following genes: *AMN*, *CD40LG*, *CD79A*, *CEACAM3*, *ESR1*, *PRL*. In Fig. 14, we used GeneMania to show that there is a relationship between the aforementioned genes, as found in the gene expression data and the literature. Blue edges represent co-localization, purple edges show co-expression, and turquoise lines show genes that belong to the same pathway.

The major genes related to top 10 diseases are represented in Table 4. Related to this table more detailed analysis for each gene is listed in Appendix: Tables 9 and 10. These tables show more details about disease associations for genes, studied country information, and genes that share more diseases with related genes.

#### Soft clustering

Soft clustering techniques are useful when items cannot be distinctly separated into clusters [8]. The clusters are formed such that each item has degrees of membership to the clusters. For example, item *A* may have a 0.1 membership value to cluster *X* and a 0.7 membership value to cluster *Y*. This technique is often used when there are items that may belong to a ‘grey’ area. We used soft clustering techniques, such as fuzzy c-means, because the separation between the clusters was not very clear (see Fig. 16). Before deciding to use fuzzy c-means, we attempted to use density-based clustering techniques, yet they were unsuccessful and only returned one cluster. We used Matlab toolbox^8^ to perform fuzzy c-means (FCM) clustering.

##### Finding the optimal number of clusters

**Fig. 15:**
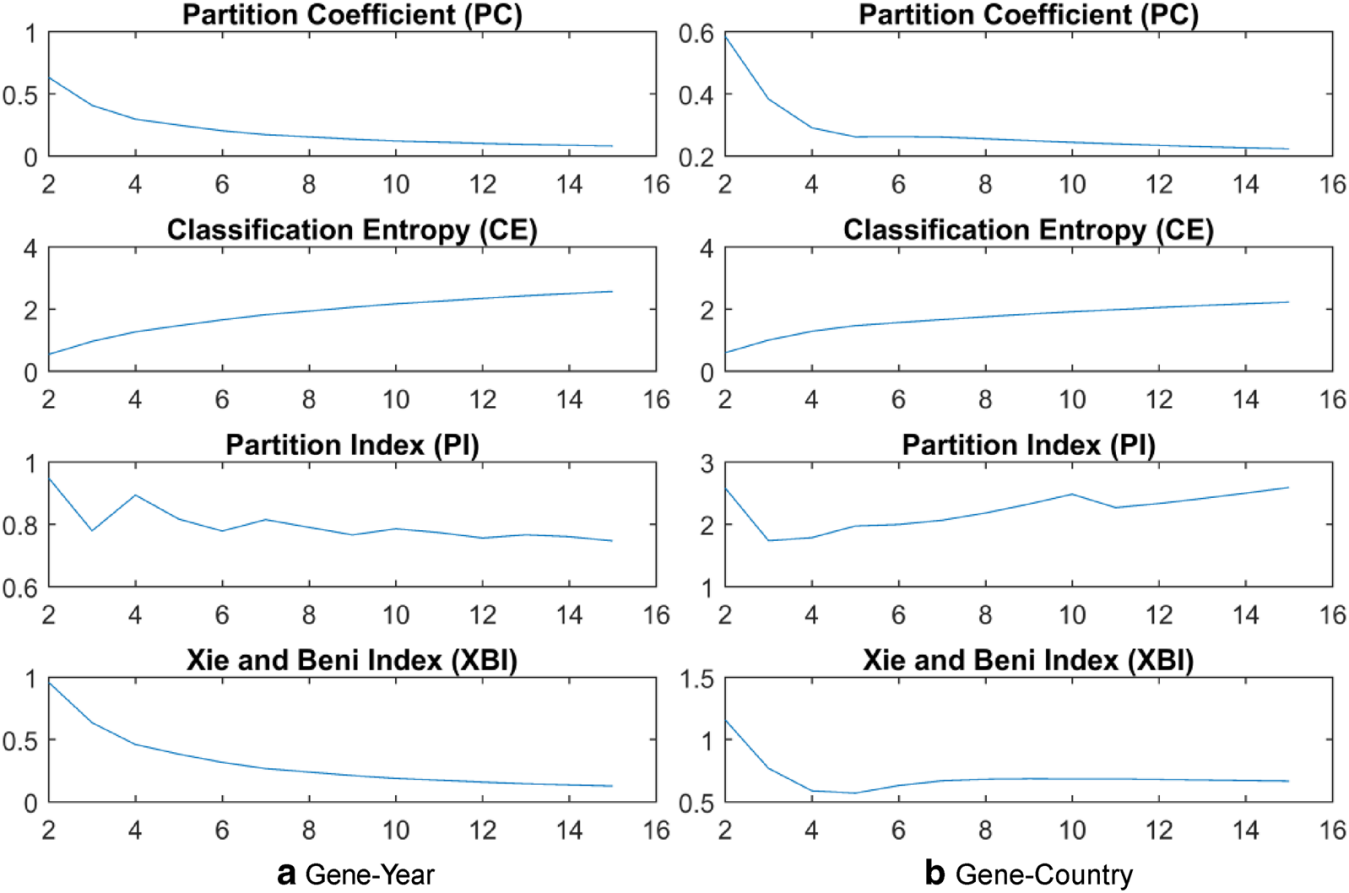
Validation of the number of fuzzy clusters using various measures

To find the optimal cluster number, we did cluster validation analysis. No validation index is reliable only by itself, so that is why all the indexes *c* (cluster numbers) between 2 and 15 are shown in Fig. 15, and the optimum can be only detected with the comparison of all the results. We consider that partitions with less clusters are better, when the differences between the values of a validation index are minor. Cluster validation is used to evaluate how well the partitions have been produced [35], which is the reason why we chose the number of clusters as 3 and 4. For the cluster validation, we used four validation indexes: partition coefficient (PC), classification entropy (CE), partition index (PI) and the Xie-Beni index (XBI).

In Fig. 15a, the main drawback of PC is that the values are monotonically decreasing as *c* increases. CE has the same problem: it monotonically increases as *c* increases, with a hardly detectable elbow point. Out of the scores for PC and CE, the number of clusters can be only rated to 3. More informative diagram is shown: PI sharply decreases at the *c* = 3 point. The XBI index is also monotonically decreasing and reaches the local minimum while *c* is increasing. Considering that PI is more useful, when comparing different validation indexes with the same *c*, we chose the optimal number of clusters as 3.

**Fig. 16:**
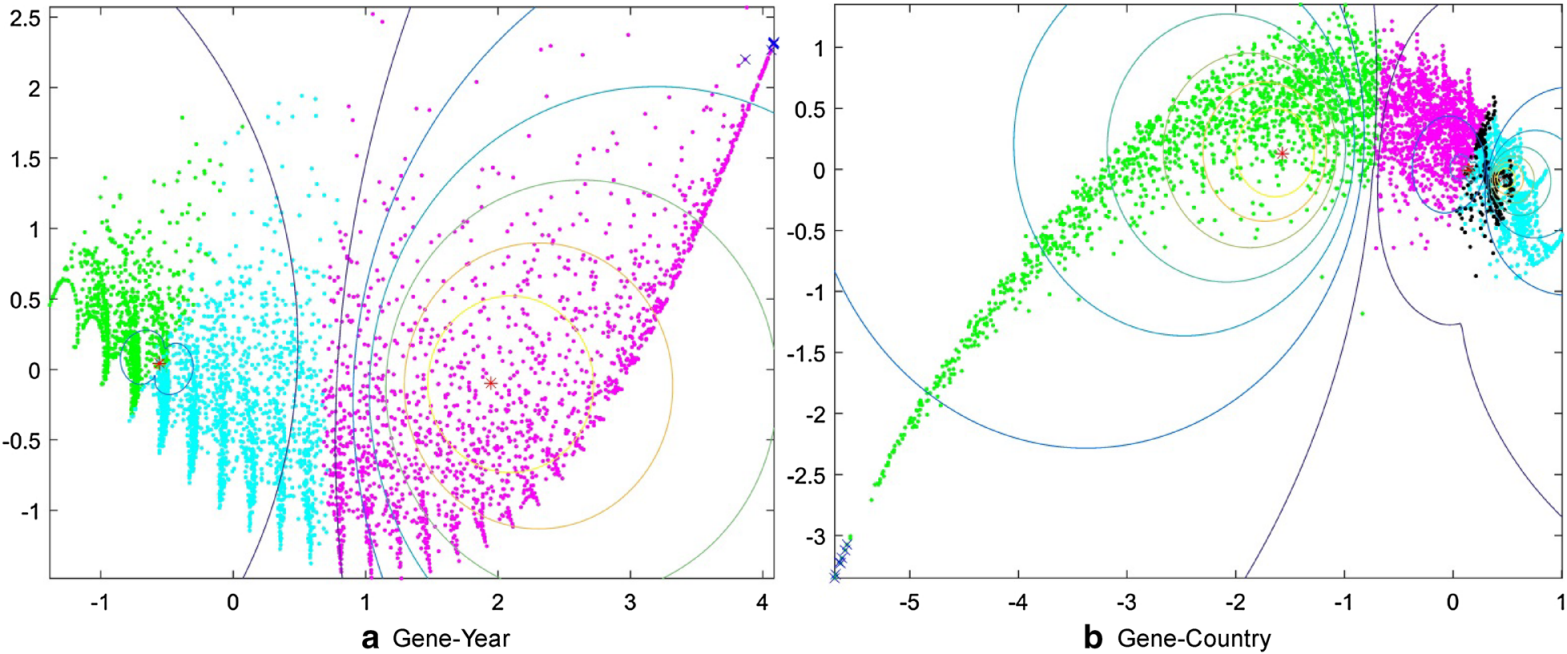
Two dimensional representations of the fuzzy clusters for the gene-year (**a**) and for gene-country (**b**) relationships. For the gene-year clusters, the number of clusters was set to 3, whereas for the gene-country clusters the number of clusters was set to 4. Each *color* represents a different cluster. *Points* marked by a *blue’x’* are the maximal closed items from the frequent mining analysis in Tables 2 and 3

In Fig. 15b, PC and CE again have the same problems: they are monotonically decreasing or increasing while *c* is increasing, which results in a hardly detectable elbow point. Out of the scores for PC and CE, the number of clusters can be only rated to 3. The more informative diagram is PI, which decreases at the *c* = 3 point. The XBI index also reaches its local minimum at *c* = 5. Considering the PI and XBI indexes, we chose the optimal number of clusters as 4. To reduce the number of dimensions to 2 (from 159 for gene-country, and 52 for gene-year) we used Principal component analysis (PCA) through Matlab in order to visualize our data (See Fig. 16).

##### Where do key genes lie in the soft clusters?

We wanted to answer the following questions: Do key genes lie in the fuzzy areas of the clusters? Did the key genes belong among different clusters? Did all the key genes belong to one cluster? We wanted to compare the results of the frequent pattern mining to that of the soft clustering.

The genes frequently mentioned together by country and year (see Tables 2, 3) which were found from a frequent mining analysis (FCM) are marked by a blue *lxl* in Fig. 16 which represents the soft clusters in 2D space. We then cross-matched the genes of the frequent pattern mining itemsets from Tables 2 and 3 with the genes of the FCM clusters. All of the genes were found to be in the fuzzy areas of the clusters, which means that none of the genes strictly belonged to one of the clusters (Fig. 16). This might mean that the genes in the closed maximal frequent item sets are key genes that are often mentioned with other genes as well across articles.

#### Network analysis

Network analysis, often called “Social Network Analysis” because it was first developed to study social structures, is a strategy to find communities within data [9]. Network analysis takes into consideration a set of “actors” and a set of “actions” between the actors. The characteristics of the actors are secondary in importance to the relationships between the actors.

There are various measures that one can use to find key actors within the network. One measure is called modularity, which is an integer that denotes what community a particular actor belongs to. Another measure is called closeness, which is a relative measure for the number of shortest paths an actor has to all other actors. The higher the closeness value that an actor has, the more connected this actor is to all other actors through short paths. In terms of sociology, an actor with a high closeness would be highly efficient at spreading information to a lot of people. A third measure that we will reference in our work is betweenness. Betweenness measures the number of shortest paths that pass through an actor. In terms of sociology, an actor with high betweenness is the best “middle man”, and if removed from the network, will disconnect a lot of people and communities.

We applied network analysis on the genes that we collected by considering the genes as “actors”, and the “actions” as co-occurrences within the abstracts. To conduct network analysis, we first built a weighted adjacency matrix between all of the genes we collected, such that each intersected value between two genes represented the number of abstracts that these two genes co-occurred within.

**Fig. 17:**
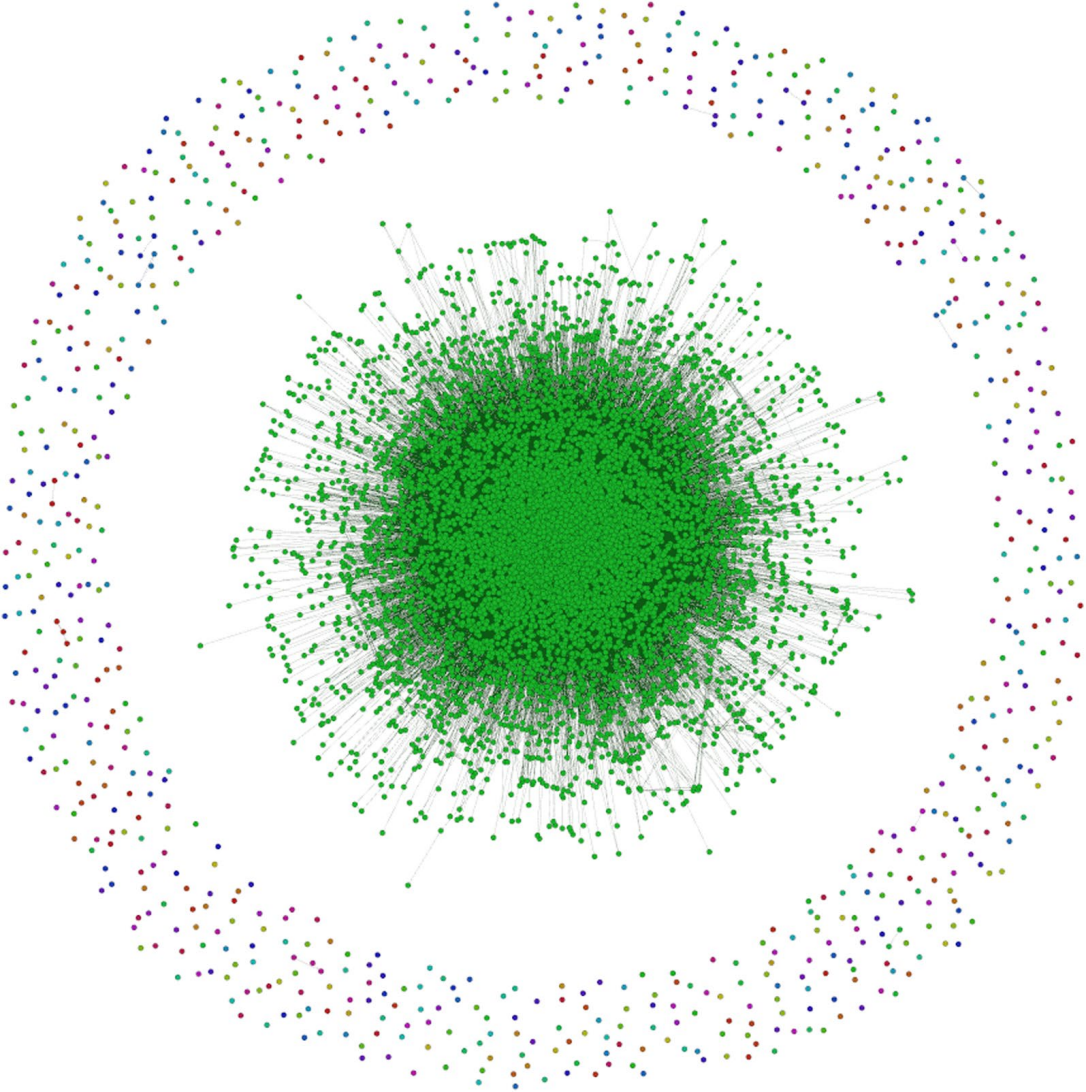
The full gene–gene network derived from the co-occurrence of genes within the abstracts. The ring of noise (disconnected genes) surrounds the network. The network is difficult to understand in this form, prior to pruning

After creating the gene–gene network from the adjacency matrix, the network contained noise comprised of some genes which were unconnected to any other genes which made it difficult to comprehend, as seen in Fig. 17. The full network contained 8400 nodes with 213,894 edges (Table 5). To get more concise results, we then did connected component analysis in order to reduce the number of edges and nodes to get the giant component. If the largest component takes a significant part of the graph, then it can be considered as the giant component [36]. Our giant component contained 90.71 % of the full network (see Table 5). However, the number of edges in the giant component, 213,877 was almost unchanged from the number of edges in the full network.

To further prepare the network for analysis, we pruned edges with weight less than 10, where edge weight is the frequency of genes’ co-occurrence in the abstracts. The pruned network was therefore more condensed and showed stronger connections, or the heart of the full network 18. To the pruned network, we applied some network measurement techniques: closeness, betweenness, and modularity. The results of the measurement are reported in Table 6, ordered by their closeness and betweenness values. Depending on these measurements, we can see the first 10 most important genes in the network, which are listed in Table 6.

In Table 6, the modularity values show which genes are making communities together, similar to clustering. For example, ESR1, ERBB2, SLC20A2, EGF, and PGR are part of the same community because they all have a modularity class of 2. To validate these results, we wanted to see if this community could also be found in experimental data. We manually validated the genes listed in Table 6 using BioGrid which is similar to GeneMania, because it uses analyzed experimental data from published articles in order to show communities of genes. We found that all genes except SLC20A2 had a physical interaction in the community. However, when we entered ESR1, ERBB2, SLC20A2, EGF, and PGR into GeneMania, it showed that all genes were indirectly related, either through shared protein domains, co-expression, pathways, etc. We, therefore, found some experimental evidence that genes in group 2 were indeed related, although the interaction may be indirect. Researchers can use these communities to find genes which may be indirectly connected, and then use experimental evidence to potentially strengthen the connection of these genes into the community.

Similarly, for genes *CDKN2A*, *BRCA1*, and *HLA-H* which all belong to modularity class 6, we performed analysis similar to that of modularity class 2. Using BioGRID, we found published evidence that CDKN2A and BRCA1 have a direct physical interaction, but not with HLA-H. However, using GeneMania, we found that there is an indirect interaction between HLA-H and the other two genes. For CDH1, we performed a different analysis, to confirm that this gene has a strong gene-disease relationship with breast cancer. We found that CDH1 has been experimentally shown to strongly influence the presence of breast cancer.^9^ For ACAD-9, we performed analysis similar to that of CDH1. To the best of our knowledge, we could not find experimental data which linked ACAD-9 to breast cancer. However, we decided to look further down the list of the most connected genes to find the next two genes which belong to class 5, so that we could perform an analysis similar to class 2 and 6. The next two well-connected genes of class 5 are MAPK10 and KRAS. GeneMania indicated that these genes are indirectly connected. Since MAPK10 codes for a protein centrally involved in a host of signalling pathways,^10^ it is likely that it is involved in cancer. Signalling proteins indicate to the cells whether they should proliferate or not, so should the protein function be defected, the cell may divide indefinitely as a cancer [34].

**Fig. 18:**
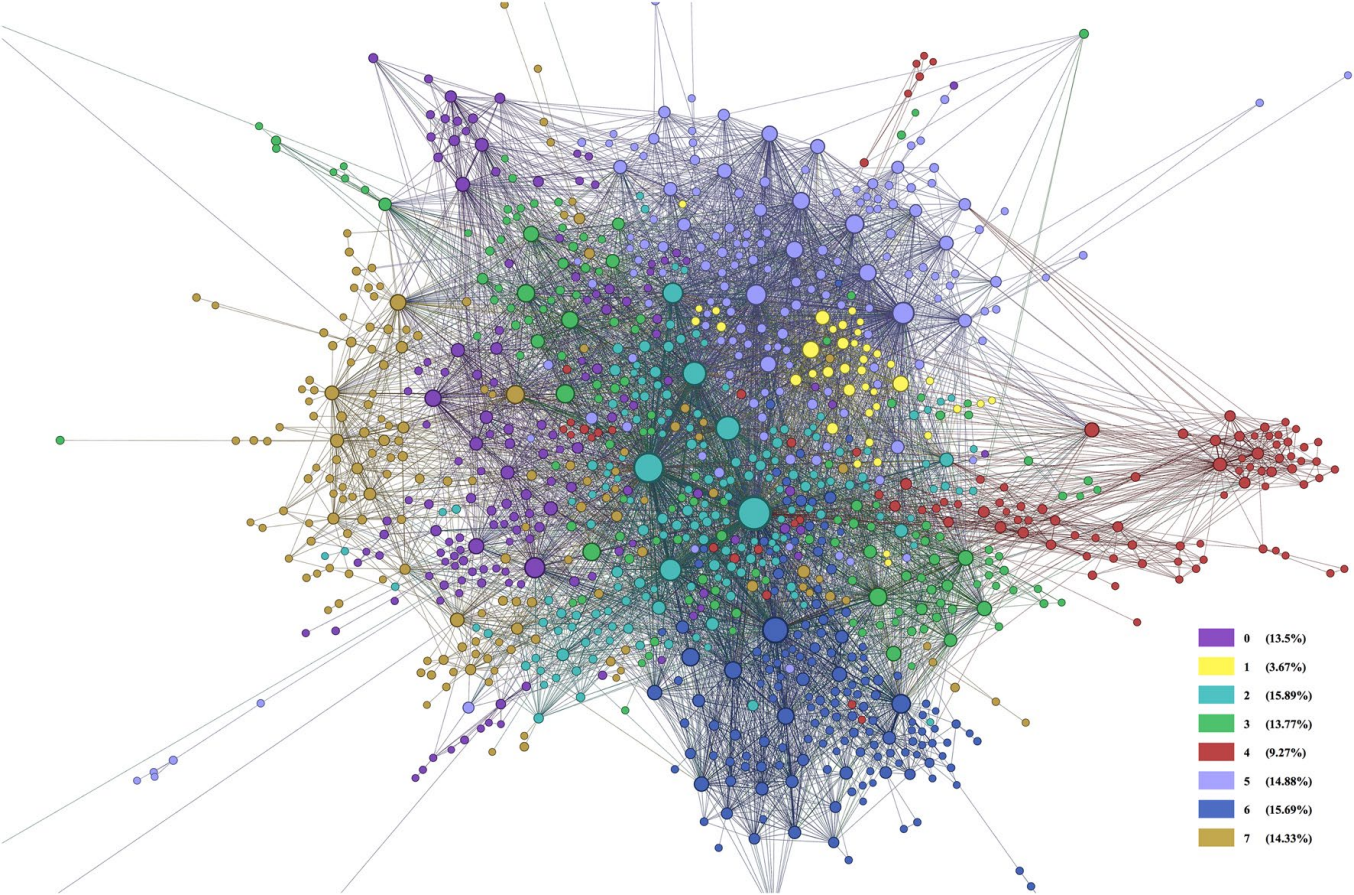
The gene–gene network. Each community is represented as a *different color*

**Fig. 19:**
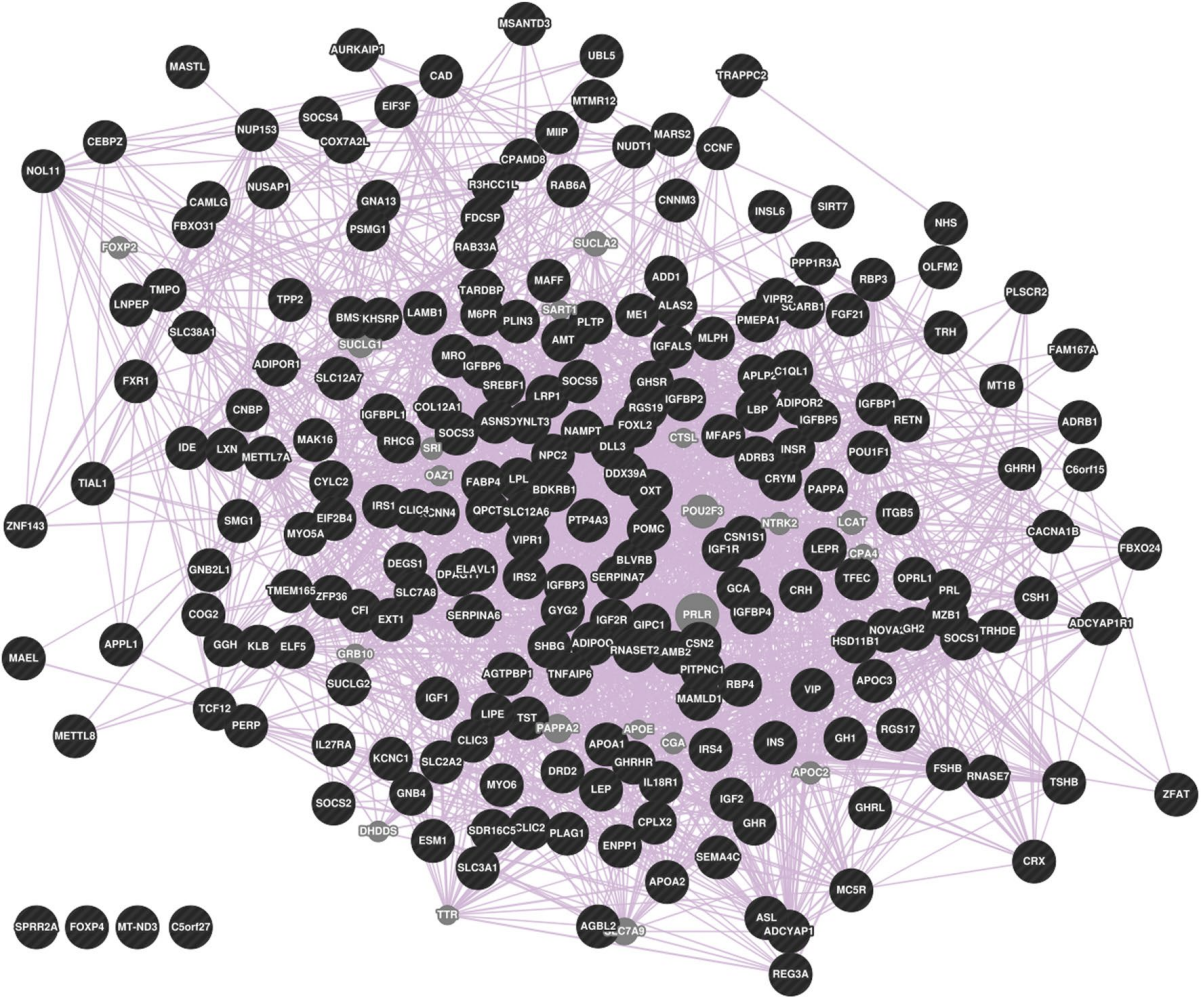
The co-expression network retrieved from GeneMania, which was used to validate the relationships between the genes within the smallest community (community 1) from our gene–gene network. Each* circle* represents a gene, and each* purple line* represents co-expression between the connected genes

We examined the smallest community (community 1 is chosen, yellow nodes in Fig. 18, which includes 229 nodes) from the pruned network to see how well the gene nodes were connected using the GeneMania resource. The results of the analysis are displayed in Fig. 19, where all genes are connected through co-expression, except for four genes: *SPRR2A*, *C5orf27*, *FOXP4*, and *MT-ND3*. The large number of connections through co-expression provides experimental support for this community. Genes which were not co-expressed with the others in the community may be genes which have yet to be validated into the community; this community may serve as a hint to primary researchers who wish to find other connections for these genes. If a researcher would like to further validate the other communities with GeneMania, we have provided the full list of network analysis genes and their modularity class (the community they belong to) in Additional file 1.

Table 7 shows which diseases are more common in each community so that we can group and target these communities based on their problem to cure. More detailed information about community-disease relation is represented in Appendix: Table 8. This table shows the top five diseases for each community and the number of genes related to each disease and the name of these genes. For example, communities 0, 2, 3, 4, and 6 are more related with cancer and its types such as breast cancer. While these communities are targeted for cancer treatment, communities 1 and 4 for diabetes mellitus, and community 7 for leukemia may be focused on treatment.

Castro et al. [37] have reported in their work that ESR1, FOXA1, GATA3, SPDEF, AR, RARA and XBP1 are critical for *ER*^+^ disease and known to be central to breast cancer risk. In our results, all these genes are found in community 2 which is the mainly related to the breast cancer, except that XBP1 is in community 3 (see Additional file 1).

## Conclusions

The work described in this paper contributes a novel framework which is capable of investigating how research groups in various countries address breast cancer. We investigated the genes or proteins studied by various research groups by carefully analyze their published research articles to identify the molecules they reported as biological biomarkers of breast cancer. Interestingly, we realized that researchers have reported interest in a variety of genes over time and even based on the country where the research is conducted. This might be due to other external factors particular and specific to each community or country, though some of the discovered genes were reported to have similar function. Thus we demonstrated how the gene–gene, gene-year, and gene-country relationships provide some interesting gene hypotheses that primary researchers might consider in their research. Further, this paper shows the power of integrating data mining and network analysis techniques.

As future work, we will also account for the semantic relations or directionality between the genes. For example, we will find relationships such as “gene A up-regulates gene B”, rather than “gene A and gene B have a relationship due to co-occurence within an abstract”. We will also attempt to upgrade the text mining application to perform full-text analysis, rather than abstract analysis. Although abstracts are useful because they summarize the articles, the full text of the articles contain more information, especially the experimental analysis and discussion sections. However, full-text mining presents many more challenges, such as errors from conversion to plain text, and problems with reading text from tables and figures [38]. We are currently investigating other types of cancer and diseases in general. We expect to report some interesting finding shortly.

## Additional file

10.1186/s13104-016-2023-5 Additional Tables.

## Appendix

See Tables 8, 9, 10 and 11.

**Table 8:** Gene-disease associations from gene–gene analysis

Community ID	Disease names	# of total genes in community	# of genes sharing disease	Gene names
0	Cancer	2105	133	EPHB4, MYCN, SOX9, RPL22, SPARC, ABL1, EAF2, PDGFA, PDGFB, SLC39A1, SPP1, RPS3, UNC5B, PIWIL1, GALR2, ETS1, DAG1, ETV4, EWSR1, CHD4, ITGA3, F2R, MMP20, ITGAV, ADAM10, ITGB3, ITGB4, TUBA4A, ZEB2, PTHLH, PTH1R, NMU, TWIST1, STRAP, JAG2, S100A4, HOXA9, BMI1, GJA1, BMP2, BMP4, BMP7, JUP, BMPR1A, JTB, CD82, HOXC8, GPC3, RHOU, NUAK1, CTNNBIP1, ITIH1, BSG, YAP1, GLI1, CTAGE1, PVRL1, KIF14, PLAU, ALAS1, MMP1, MMP2, MMP7, MMP9, MMP11, MMP14, SDC1, NANOS1, ARHGEF6, KIF11, VGF, KLK11, NID2, SFRP1, SFRP2, SFRP4, CD248, ADAMTS1, PODXL, ANXA1, USP28, WNT1, WNT2, WISP1, WNT5A, WNT7A, ARL6IP5, SLIT2, WNT2B, RIN1, SHH, GEMIN5, LAMC2, MMP26, HIF3A, RUNX2, RUNX3, KLK3, CLDN2, CLDN1, SLC2A4, ARPC2, POSTN, USP6, ORM2, HHIP, SMURF2, EFNB2, SPINT2, CD9, FAM107A, CYR61, TIMP2, TIMP3, YKT6, SNAI2, SP5, ROBO1, IRAK3, NDC80, SNAI1, CTNNB1, LUM, CTSB, KLK13, PCDH8, BCR, DKK3, RPL10, SMAD2, SMAD4, RGL4, SMAD7
	Breast cancer		65	HOXA5, WISP3, WISP2, MEST, PTPN1, HOXB13, BMP5, BMP6, UBE2B, TLK1, ETV1, KLK4, NMI, NEUROD1, ADAM28, CSF1R, PER2, RHOU, LIMD1, PTPRJ, TIMP1, ARNT2, ARID4A, TIMP4, INHBA, LATS2, TNC, USP28, SLC2A3, IGHMBP2, IBSP, VCAN, VTN, AFF3, WASF2, SERPINE1, CST3, POLI, ETS2, CSTA, LAMA3, CTGF, ADAMTS8, FURIN, MMP3, MMP8, LCN2, SIX1, MMP13, WNT9A, PCBP1, F2RL1, F3, CTSK, F7, TUBA4A, F10, SERPINA5, SDC4, RNF11, BMPR2, ANXA8L2, KLK2, PINK1, HOXA1
	Prostate cancer		63	AMBP, RNF14, KLK4, KIAA0196, PTPN1, BMP5, BMP6, BMPR1B, BMPR2, PTPN12, HOXC8, CSF1R, PDX1, EAF2, SERPINA5, PAGE4, SPINT1, SLC39A1, ACAT2, PLG, DSPP, GLI2, COPE, IBSP, VCAN, CLPTM1, EHF, SERPINE1, DVL1, ETV1, PDGFD, LATS2, CDCP1, PLAU, CRISP3, DAZL, TREX2, ELK4, TIMP1, TSPY1, RLN2, ACVR2A, CYSLTR1, ITGA7, MMP12, KLK3, MMP15, MMP17, F2RL1, ATP2A1, F3, CTSK, INHA, GFI1, HOXB13, TIMP4, RPL10, KLK2, ADAMTS9, CST3, RLN1, ZNFX1, ADAMTS13
	Diabetes mellitus		60	RLN2, XYLT2, SERPINB2, PKLR, GJA1, BMP4, BMP6, BMP7, GREM1, NEUROD1, FBP1, UTS2, CALD1, TIMP1, HLA-DMB, TIMP3, PTPRN2, SPP1, TJP1, TNC, PTX3, KCNJ10, PLA2G4A, CLPS, SERPINE1, CST3, CD9, MTTP, SHH, LRP5, ANKRD1, PTPN22, KIF11, CTGF, MMP14, GCK, ISL1, MMP1, MMP2, FTO, MMP8, TIMP2, DCN, F2, CTSB, AKR1B1, F3, ITGB3, CLOCK, AQP7, SDC2, PTGES2, SLC2A4, GGT1, FABP1, FABP2, PINK1, CYBA, SMAD7, FOXC2
	Colon cancer		51	PMP22, MMP25, RNF14, HSPE1, PTPN1, C1GALT1C1, BMPR1A, DKK4, HTR2A, CYSLTR1, STRAP, TIMP1, TIMP4, LLGL1, TJP1, TNC, ASCL2, KLF9, FDPS, TOMM34, CNOT7, ZKSCAN3, SER- PINE1, CEACAM7, SOX17, OLFM4, LYPD3, PLA2G4A, HRH2, DLL1, NTN1, ADAMTS13, MMP3, ACVR2A, LCN2, MMP10, CDCP1, MMP13, ADAMTSL3, SRPRB, F2RL1, AKR1B1, CTSH, CLDN12, ITGB6, SDC2, KLK1, GGT1, B3GNT8, CD226, ACTR2
1	Diabetes mellitus	229	29	GH1, GHR, SOCS2, NAMPT, LIPE, RETN, IGF2R, IGFBP1, IGFBP3, NUDT1, LNPEP, ADIPOR2, INS, FGF21, LPL, RBP4, POMC, APOA1, APOA2, IRS1, IDE, APOC3, HSD11B1, CFI, PLTP, LEPR, SLC2A2, ADD1, FABP4
	Obesity		23	GH1, GHSR, LIPE, IGF1, IGF2, IGFBP3, IGFBP6, ADIPOR2, INSR, SOCS3, SHBG, POMC, APOA2, IRS1, HSD11B1, RETN, SERPINA6, LEP, LEPR, SLC2A2, RBP4, FABP4, ADRB1
	Breast cancer		22	GH1, GH2, GHR, SOCS2, SLC12A6, VIP, IGF1, ADIPOQ, IGFALS, IGFBP1, TIAL1, FOXL2, INS, FBXO31, INSR, SOCS3, SHBG, SLC12A7, LEP, LEPR, ADIPOR2, IGFBPL1
	Cancer		21	IGFBP5, GHRH, GHRHR, GHSR, TPP2, TMPO, CLIC4, ELAVL1, PLAG1, PAPPA, LRP1, IRS1, PTP4A3, ADCYAP1R1, IGF1R, IGF2, IGF2R, OXT, IGFBP2, IGFBP3, IGFBP4
	Prostate cancer		17	GNA13, GHR, VIPR1, MYO6, PMEPA1, LAMB1, NUDT1, INS, SOCS3, LEP, VIP, ADIPOR2, AD- CYAP1, IGF1, ADIPOQ, IGFBP1, IGFBP2
2	Cancer	876	47	S100A2, NAT1, S100A11, NES, CDR2, ERBB2, ERBB3, ERBB4, EREG, BCL11B, MET, SCGB2A2, RARB, EGF, LRIG1, RET, NKX2-1, TK1, PA2G4, AXL, ESR1, RARRES1, CYP24A1, RBP1, PROM1, CD24, MKI67, AGR2, GATA4, ETV6, MCM2, ALCAM, MME, GPNMB, KRT7, SCEL, KIR3DL2, CTSD, TACSTD2, KIT, AKR1C1, ALK, AR, RXRA, CXADR, CCKBR, CDX2
	Breast cancer		40	NEDD8, RNF5, LHCGR, GABRP, PLAC1, CARM1, NCOR1, KRT5, CD1A, NCOA2, SCGB2A1, SCGB1D2, LATS1, RARA, SRA1, STS, MAOA, KRT18, EML4, BTC, HBEGF, TNN, CYP19A1, ESRRA, GABARAP, PIP, HTATIP2, CYP27B1, GATA3, NRG2, NCOR2, GNA12, WWTR1, NRG3, KIAA0100, F8, AKR1C2, AKR1B10, GFRA1, AREG
	Prostate cancer		28	MSR1, CARM1, NCOA2, LHB, NCOR2, NR2C1, ERBB3, RARA, STS, PELP1, HPN, CADM4, CYP19A1, ESRRA, ESRRB, HTATIP2, CYP27B1, LPXN, CYP7B1, GC, GNA12, HSD3B2, CHGA, WDR77, AR, RXRB, RXRG, AREG
	Leukemia		24	CISH, NCOA2, CKMT2, RHOH, MYH11, BCL11B, RARA, CEBPE, HOXD3, HOXD13, TLE1, GRAP2, PVRL2, GATA1, IRF8, NSD1, ETV6, GNA12, CBL, CTSG, IL2RG, ENO2, F8, SLC4A1
	Diabetes mellitus		20	IRF8, MAP4K5, HTR1A, ACTG2, ADRA2B, GAD2, BTC, GC, AR, ESR1, TRPC6, DBI, CYP24A1, AKR1B10, CHGA, CYP27B1, MME, CD24, EGF, PLXDC1
3	Cancer	1210	101	SOX2, VPRBP, ALOX12, HSP90B1, TRAF2, HIF1A, HK2, MAT2A, HSPH1, BAD, GATA6, BCL2, BCL2L1, TNFRSF10A, SPAG1, PTGS2, HNRNPK, NRP2, NRP1, TYMS, SEMA3A, TUBB3, S100P, RHBDF1, KCNA1, NDRG1, CEBPB, SPIN1, RBMX, BID, PKNOX1, FGF5, PLAGL1, KDR, BCL10, TOMM40, FGFR3, FGFR4, FH, FXYD3, EIF2AK3, HSPA5, VEGFC, MEN1, ALOX5, SDHA, SDHB, SDHC, SDHD, ALOX15B, MPG, VEGFB, CA9, IL24, VHL, SEMA4D, SFRP5, ANG, ANGPT1, ANGPT2, BCL2L10, DIABLO, ANXA2, PRDX4, L1CAM, BAG3, CASP3, ID2, BIRC2, BIRC3, XIAP, SIVA1, RELA, LYVE1, POT1, IDH1, CAV3, FIGF, TYMP, SLC2A1, CDC37, PDPN, INTS6, BNIP3, MTAP, LOX, PYCARD, NEK8, ASPH, RBM6, ALOX15, CA1, TMSB10, HSP90AA1, CTTN, ENDOG, ENG, OLIG2, BBC3, EPAS1, BIRC7
	Breast cancer		57	CRYAB, SLC6A3, PTN, ADM, TUBA1B, PARP1, KLF8, SEMA3C, TXNIP, VWF, PTPRB, CUEDC2, APLN, KLF10, IGFBP7, FCGR2A, SLC16A1, CLU, WFS1, SQSTM1, RBM3, CSNK2A1, RBMX, JAG1, MIF, TRPS1, BAK1, CASP2, MAZ, XBP1, FADS2, FGF4, CASP9, IRF1, KLF4, REL, GNA11, HES1, SMPD1, NFATC1, BCL2A1, CCL16, SLC25A5, TNFRSF10B, APOE, RRAS, IKBKB, HSF1, IL1R1, FASLG, OSGIN1, RSPO1, PLXNA1, PRDX6, LSM1, CACNA1H, BIK
	Colon cancer		41	HSPD1, AIFM1, ACSL4, TRAF1, HIP1, NOD1, CLCA1, EFNB3, SAT1, NFATC1, HTRA2, CLU, FGF20, CALR, MYOD1, HPRT1, ANXA5, MIF, PRDX1, FES, CASP6, FGF7, AATF, TMEM97, ATF3, FGF18, GLRX3, TNKS2, LCP1, HSF1, FASLG, HSPA1A, SLC16A7, FGF19, HSPA8, DDIT3, MAF, PMAIP1, KIF2C, MPG, TXN
	Diabetes mellitus		40	TXNIP, ADM, PCSK2, PARP1, HIF1A, VWF, CEBPB, ANGPT1, ANGPT2, APLN, IMPDH2, ANG, KCNJ11, SLC16A1, WFS1, CAPN1, PRDX6, KLF10, XBP1, FADS2, CASP10, SI, PLAGL1, RELA, LTBR, TNFRSF10B, APOE, PBX1, PCBD1, TNMD, ENG, HSPA1A, EIF2AK3, HSPA5, ALOX5, SLC2A1, HSPB2, CA1, KLF2, TXN
	Prostate cancer		38	HSPD1, RND3, PTN, ADM, AMD1, MAPK8IP1, TPT1, CDC37, IGFBP7, XBP1, GAPDH, SPINK1, CLU, AIFM1, SQSTM1, JAG1, CAPN1, MIF, PBX1, FGF1, LAMA5, LAMC1, FGF8, FGF9, CACNA1H, ATF3, BCL2A1, APOE, IKBKB, CHUK, PCBP2, HSPA1A, HNRNPA1, RELB, LSM1, FABP5, TXN, RPL19
4	Diabetes mellitus	601	31	GSTM1, SLC6A2, GSTP1, CYP1A1, GSTT1, MT1A, TSC22D1, ARNT, LIPC, IAPP, CETP, SLC22A4, SLC22A5, AGTR1, AGTR2, PON1, AHSG, UCP2, PYGL, CAT, REN, KEAP1, IL1RAP, ATP2A2, F5, GFPT1, EDN1, EDNRA, SOD1, SOD3, KL
	Cancer		26	GSTM1, EPHX2, GSTP1, CYP1A1, CYP1A2, CYP1B1, SLC22A18, PDCD2, TSC22D1, IAPP, PHF19, RB1, CETP, GSTT1, GLRX, FECH, AOX1, TSPO, APOBEC1, SIM2, AGPAT2, COPS2, MAP4K4, MVP, EDN2, SOD2
	Breast cancer		24	SLC22A18, PPARGC1A, CYP2B6, ARNT, CYP4Z1, PIN1, CYP21A2, INSL4, AGTR2, SLC19A3, AHR, SLCO1B3, ZFHX3, AGTR1, CAT, HSD17B1, HSD17B2, ACE, GSTO1, ATP2B2, SLC26A1, EDN1, EDNRA, SOD1
	Hypertension		21	TSPO, PPARGC1A, ACE, EPHX2, ATP2A2, GCLC, UCP2, ENPEP, SLC6A2, CAT, GSTT1, EDN1, HSD3B1, REN, CYP21A2, SLC22A2, SOD3, CFTR, AGTR1, IAPP, DBH
	Atherosclerosis		21	GSTM1, VKORC1, SOD3, PON1, AHSG, GSTO1, CYP1A1, GSTT1, GCLM, SOD1, LDLR, NR1H3, ABCC6, EDN1, KL, EDNRA, ABCD1, APOC2, AGTR1, UCP2, EPHX2
5	Cancer	988	78	EPHB2, SLC5A5, RABEP2, RHOA, RHOB, RHOC, MST1R, HRAS, RALA, RALB, WNK2, ARHGDIB, RPS6KB1, PEBP1, NEK3, PTPN6, VRK1, KRAS, JUN, MAPK14, KCNA2, PTPRA, ILK, KCNA5, AKT3, JAK2, PXN, BRAF, P2RX5, AKAP12, PTPRK, MAP2, EGR1, SDCBP, KCNH2, PIK3CG, PIK3R1, EZR, RPS6KA2, CXCL17, EIF4A2, EIF4E, DLGAP5, DAB2, IQSEC1, MAP3K1, KHDRBS1, TSC1, GPR56, TNK2, TIAM1, AKT1, AKT2, PLCB2, VAV3, PRMT3, CAV1, HBP1, GPRC5A, ARHGEF2, SNCG, MAP2K4, MELK, KISS1, GDF15, KIAA1524, SPHK1, TRIB3, RAF1, PTK2, DLC1, PKN3, CRK, RAC1, BCAR1, RAC3, LGALS7, ARF6
	Breast cancer		50	MST1, IL11RA, ADORA2B, LIMK1, EEF2, BMX, SLC9A3R1, DNAJA3, CSK, PHLDA1, IKBKE, SLC9A1, PTPRZ1, CSNK1A1, MAPKAPK2, KCNJ3, DUSP1, PDCD4, DUSP6, MBL2, EIF4EBP1, SH2D3C, EIF4G1, PAK1, ETV5, ATAD2, MLLT4, ROCK1, ACTN4, NR3C2, PLCD1, RHEB, PLD1, RB1CC1, NFATC2, EEF1D, FHL2, CHN2, RACGAP1, TSC2, TUBB, LPAR2, SH2D3A, RAB27A, RPL7A, DIRAS3, GAB2, PTK6, NEK3, WASL
	Prostate cancer		36	TYK2, FOXO1, IL11RA, PTK2B, HSPG2, SPRY2, JUND, LIMK1, SET, BMX, MAK, RAP2A, JAK1, NOX1, CSK, EGR1, F2RL3, MAPKAPK2, FDFT1, TLE3, RPS6KA3, EIF4EBP1, CPNE3, LRP2, ETV5, WFDC1, TRPM8, ELK1, PLCG1, UBIAD1, PAK6, REPS2, FHL2, LPAR1, RHEB, ITPR1
	Diabetes mellitus		31	DUSP12, EZR, GIP, ADORA2B, JUN, MAPK14, NOX1, SLC12A3, PTPRN, PIK3CG, LPA, INPPL1, EIF4A2, MBL2, LRP2, PLA2G2A, RDX, AKT1, AKT2, RORC, LRRC7, EIF4E, ARHGEF11, CHRM3, ELMO1, ITPR3, MAP3K1, CRTC2, EXOC4, MSN, TRPC1
	Rheumatoid arthritis		24	SLC5A5, RHOA, JAK2, JUN, MAP2K4, MRAS, NEDD9, BMX, PIK3CG, MAPK14, CENPJ, CSK, EGR1, IKBKE, GDF15, TRPC1, MBL2, EIF4G1, LRP2, C5, LPAR1, GAB2, RAC1, MAP3K2
6	Cancer	1397	122	MYC, CDKN1A, CDKN1B, CDKN1C, CDKN2A, CDKN2B, CDKN2C, SP1, SP4, PTTG1, ERCC1, ERCC2, CEBPA, ERH, ATR, STAG1, XRCC1, TRIO, HDAC8, PPP1R13L, BARD1, DCK, NBN, MCM3, EZH2, MCM7, CCND1, CAGE1, CHEK1, ALDH1L1, DCC, RRM1, RRM2, MDM4, ID4, ECT2, GADD45A, MOAP1, TUBG1, RYR1, DDX5, MAP3K4, NIT2, ADH1B, ADH1C, AQP1, HDAC3, CKS1B, FAP, RPRM, MGMT, BRCA1, BRCA2, KCNH1, TMPRSS2, SUPT7L, BUB1, MLH1, CDC73, FHIT, MBD4, PLK1, COPS5, SMYD3, BRMS1, RAD51, FOXM1, PMS2, BCL2L15, HDAC5, RBBP4, NEIL1, RBL2, UBE2C, APC, SHMT1, APEX1, RECQL, E2F2, E2F3, MSH2, LASP1, RNF139, NEK2, XRCC3, SKP2, IGF2BP1, ASH2L, PDLIM5, CCNA2, CCNB1, CCND2, CCND3, CCNE1, CCNG1, MSH6, TRAF4, IGF2BP3, MTA1, RNF2, RFWD2, MTHFR, EPHA2, WIF1, FBXO4, CST6, EXO1, SMARCA4, SMARCB1, DAPK1, RPL11, E2F1, ATM, FSCN1, PUM1, SH2D1A, MUTYH, MAD2L1, PCNA, XPC, AURKB, MYBL2
	Breast cancer		64	CDK9, RAD52, TOPBP1, FANCD2, MRE11A, HSPB8, MYBBP1A, RPS6KA6, BCAS2, ERCC4 RNASEL, CEBPD, HDAC6, HDAC4, XRCC2, DERL1, NOL3, MUS81, CENPF, CTCF, INHBB, RBBP7, RBBP8, RBL1, CCNE2, POLB, KLF5, C1QA, WWP1, XRCC4, CAPN2, PRC1, PEMT, MED14, PAK2, BCCIP, MTRR, XDH, SMARCE1, FOXP1, SH3GL2, E2F4, NCL, PBOV1, ANXA8, RRAD, SIPA1, CHKA, ATP1B2, MBD2, NOD2, PRDM14, DDB2, DUSP22, RGS2, PALB2, EP300, CLSPN, HIST2H3A, MLLT11, RAD50, RAD17, KPNA2, RAD23B
	Prostate cancer		41	GLIPR1, SUMO1, ERCC1, MT2A, IRX5, RNASEL, RCHY1, CEBPD, ERG, PALB2, BRCA2, PI16, BTRC, LZTS1, RBL1, KLF5, SGTA, TSGA10, SMARCA2, CCNA1, PAK2, SMARCC1, MTRR, FOXP1, TSG101, MSH3, PBOV1, RPS27A, TOPORS, SENP1, NUPR1, AQP3, CREBBP, MECP2, MSMB, ELAC2, EP300, CDK5R1, RAD9A, PCNT, RAD21
	Colon cancer		39	BLM, CITED2, SLC6A4, MRE11A, SND1, CDX1, MLH3, DDX17, HTR3A, LMNA, POLD1, CENPA, NOL3, UCHL1, NEIL2, KLF5, MATK, BRD7, TSGA10, RPS6KA6, HLTF, BCAT1, BCHE, CTBP1, E2F4, XPA, MSH3, LTC4S, CDC16, CHKA, CD3EAP, AIM2, METAP2, EP300, PPM1H, DDX5, RAD18, NOD2, CA8
	Embryoma		34	BLM, PCSK1, AVEN, HDAC11, HOXC9, RNASEL, RPRM, RNF2, HTR3A, FBXO4, STAG1, RCHY1, BCAS2, KLF5, UCHL1, WWP1, GAS1, ASS1, MTR, UHRF1, RECQL, NCL, XPO1, CCL23, CBS, MECP2, HDAC8, PALB2, RPL11, MAP3K4, HMG20B, DNMT3L, PCNT, KPNA2
7	Leukemia	967	77	MPO, IFNG, CXCR5, IL11, SELE, RAG1, RAG2, SELL, ITGAX, ORM1, IL10, KLRC1, CD2, CD52, IL18, CD5, CSF1, CD7, CD8A, CSF3, CSF3R, CD19, P2RX7, CIITA, CALCA, ASAH1, CD86, TN- FRSF8, CD33, IGHM, CCL21, B2M, PVR, IL21, TNF, LAIR1, CCL2, CD160, HLA-A, ULBP2, CCL3, ICAM1, LAMP1, HLA-B, CCL4, CCL5, CCR4, GNLY, KIR3DL1, CCL11, CTLA4, CCL18, GCNT1, CCL19, ITGA4, CHIT1, CCL22, IL1A, IL1B, ITGAL, ITGAM, LYZ, IL2, IL2RA, IL2RB, IL3RA, TTR, IL4, IFNA1, CD83, IL6, IL7, SPANXB1, BGN, PML, PDCD1LG2, FAIM3
	Rheumatoid arthritis		70	SELE, OSM, DEFA1, TPSAB1, ADORA3, IL15, IL16, TNFRSF9, IL17A, MAL, MGAT5, CD5, CSF1, KIR2DL1, TIA1, CD14, HPSE, HLA-C, FCGR3A, SELP, ITGA4, HRH4, CD80, CD86, TNFRSF8, ACP5, ICAM1, ICAM3, IL21, MITF, CCL18, IL1A, LAMP3, CXCL13, CD274, MDK, CCRL2, ICOS, CCL3, IRF3, CCR2, CCL5, LTA, CCL3L1, P2RX7, CCL11, CCL13, TLR2, C5AR1, HAMP, GCNT1, CXCL16, CHI3L1, LTB4R2, TNFRSF17, IL1B, IL2, XCL1, CX3CL1, ITGB2, CCL20, IL4, CD276, CD83, CXCL12, IL7, TNF, PML, PDCD1LG2, LGALS9
	Prostate cancer		59	ARG2, IFNB1, A2M, IL10RA, MAGEA1, MAL, MAGEA4, S100A9, CXCL10, IFNG, IL15, IL16, IL18, TES, MGAT5, CSF1, CALCA, CALCR, HLA-A, ASAH1, MPO, SEC62, AGER, IL10, AZGP1, ITGA5, ICAM1, TLR1, TLR3, MCAM, CCL2, CD55, STEAP2, B2M, CCR1, CCR2, CCL5, CCR9, TNF, CTLA4, CRP, ITGA2, GCNT1, CXCL16, CHI3L1, TLR6, CHIT1, S100A8, OSM, LCT, IL1RN, IL2, CSMD1, IL4, IL6, ACPP, PML, PTMA, RING1
	Diabetes mellitus		57	MPO, IFNG, DEFA1, SELE, EPO, IL13, SELL, IL15, SELP, CD4, ITGA2B, CSF3, HLA-A, LCAT, HP, CD86, AGER, GLP1R, ICAM1, TLR3, IL21, P2RX7, CMA1, MDK, MCAM, CD55, HRH4, CCL2, CASQ1, CCR2, CCL5, LTA, ALAD, GNAI2, TNF, CTLA4, GGT2, ITGA2, GCNT1, KIR2DL2, IL1A, ITGAM, HPSE, ITGB2, TTR, IL4, IFNA1, MEF2C, PCK1, CXCL12, CD163, LGALS3, BGLAP, CRP, MC3R, TNFRSF4, APOC1
	Cancer		55	MAGEA3, CCNT1, EPOR, IL13RA2, AMPH, SERPINB4, CEACAM5, KITLG, GALNT3, FCER2, ANPEP, MS4A1, SPN, PDZK1IP1, NCR2, CD99, AFP, EPO, CD34, THY1, CAPG, CYP27A1, VTCN1, TIA1, C1QBP, CEACAM6, CXCL14, ST3GAL6, EBAG9, HPSE2, CCR3, ST3GAL4, PAX5, ATOH1, STIL, BCL6, CASC5, MDK, PBX2, CTSE, MUC2, SLAMF1, ST18, IL3, HPSE, MUC6, HNRNPF, CXCL12, LGALS1, LGALS3, SLC3A2, CD200, CEACAM1, TPD52, FGFBP1

**Table 9:** Gene-disease associations from gene-year and gene-country analysis

Gene	Disease associations for gene		Genes that share more diseases with this gene		Country associations for gene	
	Disease name	Score	Gene name	# of shared diseases	Country name	# of abstracts
ERBB2	Breast neoplasms	0.414	EGFR	15	United States	4271
	Mammary neoplasms, experimental	0.4	PTGS2	13	Italy	808
	Neoplasm metastasis	0.396	SOD2	12	Japan	806
	Adenocarcinoma	0.363	TP53	11	China	799
	Ovarian neoplasms	0.331	STAT3	10	United Kingdom	674
	Prostatic neoplasms	0.329	CCND1	10	Germany	620
	Lung neoplasms	0.329	ESR1	10	France	486
	Stomach neoplasms	0.321	KRAS	9	Canada	433
	Cholangiocarcinoma	0.308	TNF	9	South Korea	347
	Glioma	0.306	TNFSF10	9	Spain	329
ESR1	Breast neoplasms	0.423	SOD2	14	United States	5429
	Alzheimer disease	0.358	EGFR	13	United Kingdom	1249
	Neoplasm metastasis	0.345	PTGS2	12	Japan	918
	Carcinoma	0.344	TNF	11	China	764
	Coronary artery disease	0.342	CDH1	10	Italy	727
	Migraine disorders	0.333	ACE	10	France	569
	Obesity	0.327	ERBB2	10	Germany	517
	Leiomyoma	0.327	PTEN	9	Canada	515
	Myocardial infarction	0.323	STAT3	9	South Korea	338
	Infertility, male	0.321	TP53	9	Sweden	299
PGR	Breast neoplasms	0.38	EGFR	7	United States	1887
	Endometriosis	0.346	ESR1	6	Japan	456
	Carcinoma	0.32	ESR2	6	Italy	404
	Meningioma	0.307	STAT3	5	China	385
	Adenocarcinoma	0.304	EFEMP1	5	United Kingdom	311
	Mammary neoplasms, animal	0.3	CDH1	5	France	294
	Mammary neoplasms, experimental	0.3	PHB	5	Germany	245
	Mesothelioma	0.3	PDGFA	5	Canada	188
	Recurrence	0.3	STAT5A	5	South Korea	169
	Malignant neoplasm breast	0.126	ENO1	5	Sweden	131
EGF	Hypomagnesemia 4, renal	0.6	SOD2	11	United States	2199
	Wounds and injuries	0.4	IL6	9	United Kingdom	408
	Breast neoplasms	0.325	MMP9	9	Japan	394
	Prostatic neoplasms	0.322	PTGS2	9	China	378
	Carcinoma, hepatocellular	0.317	TNF	9	Italy	298
	Neoplasm metastasis	0.315	PTEN	8	Germany	239
	Glioblastoma	0.311	EGFR	8	South Korea	211
	Adenocarcinoma	0.307	IGF1	8	Canada	205
	Kidney diseases	0.301	IL8	8	France	173
	Stomach ulcer	0.3	TGFB1	7	Spain	112
BRCA1	Breast-ovarian cancer, familial, Susceptibility To, 1	0.7	CDH1	7	United States	1845
	Malignant neoplasm breast	0.54	CCND1	7	United Kingdom	395
	Malignant neoplasm of ovary	0.44	SOD2	7	Canada	304
	Breast neoplasms	0.419	BRCA2	6	France	222
	Mammary neoplasms, experimental	0.4	HRAS	6	The Netherlands	218
	Ovarian neoplasms	0.381	STAT3	6	Italy	197
	Neoplasms	0.375	EGFR	6	China	182
	Carcinoma	0.366	ERBB2	6	Spain	143
	Hereditary breast and ovarian cancer Syndrome	0.359	ESR1	6	Germany	140
	Prostatic neoplasms	0.318	AKT1	5	Japan	124
BRCA2	Fanconi anemia, complementation Group D1	0.7	BRCA1	6	United States	885
	Malignant neoplasm breast	0.54	CTNNB1	6	United kingdom	256
	Ovarian neoplasms	0.464	ERBB2	6	Canada	203
	Prostatic neoplasms	0.409	PTEN	5	Italy	119
	Medulloblastoma	0.401	SOD2	5	The Netherlands	115
	Breast neoplasms	0.392	TNF	5	Germany	102
	Hereditary breast and ovarian cancer Syndrome	0.334	TNFSF10	5	France	100
	Fanconi ANEMIA	0.326	AKT1	4	Spain	93
	Pancreatic neoplasms	0.309	BRIP1	4	Australia	73
	Wilms tumor	0.3	CDH1	4	Israel	70
CDKN2A	Melanoma-pancreatic cancer syndrome	0.6	TP53	15	United States	1809
	Melanoma, cutaneous malignant, susceptibility To, 2	0.6	SOD2	12	China	431
	Lung neoplasms	0.442	KRAS	9	Japan	340
	Stomach neoplasms	0.411	PTGS2	9	United Kingdom	325
	Esophageal neoplasms	0.41	ABCB1	7	Italy	297
	Neoplasms	0.391	CSF3	7	France	224
	Adenocarcinoma	0.358	EGFR	7	Germany	218
	Glioma	0.341	ESR1	6	South Korea	202
	Precursor cell lymphoblastic leukemia-Lymphoma	0.338	MET	6	Canada	186
	Carcinoma, non-small-cell lung	0.332	ERBB2	6	Spain	136
ALPPL2	Abortion, spontaneous	0.3	CEACAM1	1	United States	104
	Parkinson disease	0.003	HSD17B1	1	Germany	32
	Stroke	0.003	IFI35	1	United Kingdom	30
	Carcinoma in situ	0.001	IFI44	1	Italy	28
	Seminoma	0.001	IFI6	1	France	18
	Retinal diseases	<0.001	IFNA10	1	Japan	16
	Embryonal neoplasm	<0.001	IGFBP1	1	Canada	13
	Carcinoma, embryonal	<0.001	IGFBP6	1	Greece	11
	–	–	IL11	1	The Netherlands	9
	–	–	IL12B	1	China	9
CD99	Chondrosarcoma, mesenchymal	0.3	PDGFRA	1	United States	342
	Neuroectodermal tumors, primitive, Peripheral	0.012	BCL2	1	Japan	77
	Sarcoma, ewing	0.01	IL1A	1	Germany	74
	Breast neoplasms	0.005	MKI67	1	Italy	63
	Carcinoma	0.005	–	–	China	56
	Neuroectodermal tumors, primitive	0.004	–	–	United Kingdom	52
	Osteosarcoma	0.003	–	–	Canada	37
	Neoplasms	0.003	–	–	France	26
	Lymphoma	0.003	–	–	Australia	24
	Adenocarcinoma	0.003	–	–	The Netherlands	22
CHI3L1	Schizophrenia	0.319	TNF	3	United States	90
	Glioblastoma	0.311	MET	3	Japan	17
	Glioma	0.31	MGMT	2	United Kingdom	17
	Neoplasm invasiveness	0.303	TGM2	2	Italy	15
	Osteoarthritis	0.301	ACO1	2	France	12
	Asthma-related traits, susceptibility To, 7	0.3	MMP9	2	Denmark	10
	Hypertension	0.103	GDNF	2	Germany	6
	Asthma	0.017	FTL	2	Australia	6
	Arthritis, rheumatoid	0.009	ENO1	2	Finland	5
	Neoplasm malignant	0.005	EGF	2	India	5
SOD1	Amyotrophic lateral sclerosis 1	0.66	TNF	19	United States	265
	Amyotrophic lateral sclerosis	0.551	SOD2	17	Italy	42
	Hypertension	0.402	IL6	15	Japan	40
	Deficiency diseases	0.4	PTGS2	14	India	39
	Motor neuron disease	0.341	NOS2	13	China	31
	Down syndrome	0.323	CAT	13	United Kingdom	28
	Atherosclerosis	0.31	AGT	11	Germany	26
	Diabetes mellitus, type 2	0.31	IL1B	10	The Netherlands	25
	Ischemia	0.309	IFNG	10	Turkey	20
	Parkinson disease	0.309	ALB	10	Canada	18
AMN	Imerslund-grasbeck syndrome	0.601	TNF	3	United States	138
	Acute kidney injury	0.3	KNG1	3	United Kingdom	32
	Neurogenic inflammation	0.3	TAC1	3	Germany	22
	Edema	0.3	IL6	2	Japan	18
	Extravasation of diagnostic and Therapeutic Materials	0.3	POMC	2	China	16
	anemia, megaloblastic	0.003	CALCA	2	Canada	12
	adrenoleukodystrophy	0.003	PTGS2	2	France	10
	Nervous system malformations	0.003	INS	2	Italy	9
	Malabsorption syndromes	0.001	KLK1	1	The Netherlands	9
	Adrenomyeloneuropathy	<0.001	LCN2	1	Australia	7
CD40LG	Hyper-igm immunodeficiency syndrome, Type 1	0.629	CCL2	4	United States	66
	Coronary artery disease	0.306	IL1B	3	Germany	15
	Pneumonia	0.3	IL6	3	United Kingdom	12
	Amyotrophic lateral sclerosis	0.3	TNF	3	Japan	11
	Hypersensitivity	0.3	IL8	3	Italy	10
	Necrosis	0.3	IFNG	3	Argentina	9
	Hypertension, pulmonary	0.3	IL5RA	2	China	9
	Diabetes mellitus, type 1	0.101	HMOX1	2	Australia	7
	Enterocolitis, necrotizing	0.1	IL13	2	Denmark	6
	Periodontal diseases	0.1	IL17A	2	The Netherlands	6
CD79A	Agammaglobulinemia	0.3	BTK	1	United States	22
	Leukemia, lymphocytic, chronic, B Cell	0.003	CD19	1	France	8
	Lymphoma, non-hodgkin	0.003	IGLL1	1	China	7
	Lymphoma, B-cell	0.003	LRRC8A	1	Japan	6
	Leukemia, myeloid, acute	0.003	–	–	India	4
	Leukemia	0.003	–	–	Spain	4
	Multiple myeloma	0.003	–	–	Sweden	3
	Lymphoma	<0.001	–	–	Belgium	3
	Takayasu arteritis	<0.001	–	–	Finland	3
	Lymphoma, large B-Cell, diffuse	<0.001	–	–	United Kingdom	3
PRL	Prolactinoma	0.415	DRD2	9	United States	294
	Hyperprolactinemia	0.412	POMC	8	United Kingdom	59
	Adenoma	0.33	IL6	6	Italy	55
	Lupus erythematosus, systemic	0.325	CYP19A1	6	Canada	40
	Pituitary neoplasms	0.311	TNF	6	France	31
	Autistic disorder	0.304	ESR2	5	Australia	29
	Growth hormone-secreting pituitary Adenoma	0.302	AGT	5	Japan	28
	Endometriosis	0.301	CNR1	5	China	23
	Hypopituitarism	0.301	CRH	5	Spain	20
	Amenorrhea	0.301	CYP17A1	5	India	19
AFP	Carcinoma, hepatocellular	0.398	MMP9	5	United States	46
	Liver diseases	0.303	HMOX1	4	Japan	12
	Liver cirrhosis, experimental	0.3	ENO1	3	China	11
	Breast neoplasms	0.3	MMP2	3	Germany	8
	Mammary neoplasms, experimental	0.3	ESR1	3	Italy	7
	Liver neoplasms	0.019	HRAS	3	France	6
	Recurrent malignant neoplasm	0.015	NOS2	3	Canada	5
	Hepatitis B	0.014	IGF1	3	Ireland	4
	Neoplasm malignant	0.012	PTGS2	3	Turkey	3
	Down syndrome	0.011	TNFSF10	3	Singapore	3
POMC	Obesity	0.454	TNF	22	United States	27
	Proopiomelanocortin deficiency	0.4	IL6	17	Italy	10
	Cushing syndrome	0.331	AGT	15	Japan	9
	Pituitary acth hypersecretion	0.315	IL1B	15	France	7
	Adrenal cortex diseases	0.309	PTGS2	15	United kingdom	6
	Acth syndrome, ectopic	0.306	SOD2	14	Spain	5
	Heart failure	0.304	ALB	12	The netherlands	5
	Spasms, infantile	0.303	INS	12	Germany	4
	Hypertension	0.303	BDNF	11	Austria	4
	Osteoporosis	0.303	CRH	11	Poland	4

**Table 10:** Genes are related to diseases depends on gene-year and gene-country analysis

	ERBB2	PRL	CD79A	CD40LG	AMN	SOD1	CHI3L1	AFP	CD99	CDKN2A	BRCA2	BRCA1	EGF	PGR	ESR1	POMC	ALPPL2
Breast neoplasms	X							X	X		X	X	X	X	X		
Adenocarcinoma	X								X	X			X	X			
Mammary neoplasms, experimental	X							X				X		X			
Carcinoma									X			X		X	X		
Prostatic neoplasms	X										X	X	X				
Malignant neoplasm breast											X	X		X			
Glioma	X						X			X							
Hypertension						X	X								X		
Neoplasms									X	X		X					
Ovarian neoplasms	X										X	X					
Neoplasm metastasis	X												X		X		
Down syndrome						X		X									
Lymphoma			X						X								
Glioblastoma							X						X				
Carcinoma, hepatocellular								X					X				
Hereditary breast and ovarian cancer syndrome											X	X					
Endometriosis		X												X			
Parkinson disease						X										X	
Neoplasm malignant							X	X									
Obesity															XX		
Amyotrophic lateral sclerosis				X		X											
Coronary artery disease				X											X		
Lung neoplasms	X									X							
Stomach neoplasms	X									X							
Agammaglobulinemia			X														
Periodontal diseases				X													
Enterocolitis, necrotizing				X													
Diabetes mellitus, type1				X													
Hypertension, pulmonary				X													
Necrosis				X													
Hypersensitivity				X													
Imerslund-Grasbeck syndrome					X												
Hyper-Igm immunodeficiency syndrome, type 1				X													
Edema					X												
Adrenomyeloneuropathy					X												
Acute kidney injury					X												
Malabsorption syndromes					X												
Nervous system malformations					X												
Adrenoleukodystrophy					X												
Leukemia, lymphocytic, chronic, B-cell			X														
Neurogenic inflammation					X												
Extravasation of diagnostic and therapeutic materials					X												
Pneumonia				X													
Anemia, megaloblastic					X												
Adenoma		X															
Lymphoma, B-cell			X														
Heart failure															X		
Acth syndrome, ectopic															X		
Adrenal cortex diseases															X		
Pituitary acth hypersecretion															X		
Cushing syndrome															X		
proopiomelanocortin deficiency															X		
Hepatitis B								X									
Recurrent malignant neoplasm								X									
Liver neoplasms								X									
Liver cirrhosis, experimental								XX									
Liver diseases								X									
Lymphoma, non-hodgkin			X														
Amenorrhea		X															
Growth hormone-secreting pituitary adenoma		X															
Autistic disorder		X															
Pituitary neoplasms		X															
Lupus erythematosus, systemic		X															
Hyper prolactinemia		X															
Prolactinoma		X															
Lymphoma, large B-cell, diffuse			X														
Takayasu Arteritis			X														
Multiple myeloma			X														
Leukemia			X														
Leukemia, myeloid, acute			X														
Hypopituitarism		X															
Ischemia						X											
Asthma-related traits, susceptibility to, 7							X										
Atherosclerosis						X											
Wilms tumor											X						
Pancreatic neoplasms											X						
Fanconi anemia											X						
Medulloblastoma											X						
Fanconi anemia, complementation group D1											X						
Malignant neoplasm of ovary												X					
Breast-ovarian cancer, familial, susceptibility to, 1												X					
Stomach ulcer													X				
Kidney diseases													X				
Wounds and injuries													X				
Hypomagnesemia 4, renal													X				
Recurrence														X			
Mesothelioma														X			
Mammary neoplasms, animal														X			
Meningioma														X			
Infertility, male															X		
Myocardial infarction															X		
Leiomyoma															X		
Migraine disorders															X		
Alzheimer disease															X		
Cholangio carcinoma	X																
Melanoma-pancreatic cancer syndrome										X							
Melanoma,CutaneousMalignant,SusceptibilityTo,2										X							
Esophageal neoplasms										X							
Precursor cell lymphoblastic leukemia-lymphoma										X							
Motor neuron disease						X											
Deficiency diseases						X											
Amyotrophic lateral sclerosis1						X											
Arthritis, rheumatoid							X										
Asthma							X										
Spasms, infantile																X	
Osteoarthritis							X										
Neoplasm invasiveness							X										
Schizophrenia							X										
Osteosarcoma									X								
Diabetes mellitus, type 2						X											
Neuroectodermal tumors, primitive									X								
Neuroectodermal tumors, primitive, peripheral									X								
Chondrosarcoma, mesenchymal									X								
Carcinoma, embryonal																	X
Embryonal neoplasm																	X
Retinal diseases																	X
Seminoma																	X
Carcinoma in situ																	X
Stroke																	X
Abortion, spontaneous																	X
Carcinoma, non-small-cell lung										X							
Sarcoma, ewing									X								
Osteoporosis																X

**Table 11:** Top 10 genes are mentioned by each country

Country name	# of abstracts	Gene name		Country name	# of abstracts	Gene name	
United States	33,373	ESR1	5429 [16.27 %]	Germany	4148	ERBB2	620 [14.95 %]
		ERBB2	4271 [12.8 %]			ESR1	517 [12.46 %]
		EGF	2199 [6.59 %]			PGR	245 [5.91 %]
		PGR	1887 [5.65 %]			EGF	239 [5.76 %]
		BRCA1	1845 [5.53 %]			CDKN2A	218 [5.26 %]
		CDKN2A	1809 [5.42 %]			SLC20A2	191 [4.6 %]
		SLC20A2	1418 [4.25 %]			BRCA1	140 [3.38 %]
		TKT	1297 [3.89 %]			CYP19A1	120 [2.89 %]
		ACAD9	1143 [3.42 %]			KRT75	120 [2.89 %]
		CYP19A1	1073 [3.22 %]			TKT	116 [2.8 %]
United Kingdom	6041	ESR1	1249 [20.68 %]	France	3642	ESR1	569 [15.62 %]
		ERBB2	674 [11.16 %]			ERBB2	486 [13.34 %]
		CYP19A1	425 [7.04 %]			PGR	294 [8.07 %]
		EGF	408 [6.75 %]			CDKN2A	224 [6.15 %]
		BRCA1	395 [6.54 %]			BRCA1	222 [6.1 %]
		CDKN2A	325 [5.38 %]			EGF	173 [4.75 %]
		PGR	311 [5.15 %]			SLC20A2	165 [4.53 %]
		BRCA2	256 [4.24 %]			TKT	131 [3.6 %]
		SLC20A2	227 [3.76 %]			CYP19A1	120 [3.29 %]
		INS	188 [3.11 %]			CTSD	114 [3.13 %]
China	6553	ERBB2	799 [12.19 %]	Canada	3573	ESR1	515 [14.41 %]
		ESR1	764 [11.66 %]			ERBB2	433 [12.12 %]
		CDKN2A	431 [6.58 %]			BRCA1	304 [8.51 %]
		PGR	385 [5.88 %]			EGF	205 [5.74 %]
		EGF	378 [5.77 %]			BRCA2	203 [5.68 %]
		ACAD9	336 [5.13 %]			PGR	188 [5.26 %]
		MYLIP	327 [4.99 %]			CDKN2A	186 [5.21 %]
		BCL2	312 [4.76 %]			INS	146 [4.09 %]
		ABCB1	209 [3.19 %]			TKT	137 [3.83 %]
		CASP3	203 [3.1 %]			SLC20A2	136 [3.81 %]
Japan	5299	ESR1	918 [17.32 %]	The Netherlands	1844	ESR1	267 [14.48 %]
		ERBB2	806 [15.21 %]			BRCA1	218 [11.82 %]
		PGR	456 [8.61 %]			ERBB2	181 [9.82 %]
		EGF	394 [7.44 %]			BRCA2	115 [6.24 %]
		CDKN2A	340 [6.42 %]			PGR	115 [6.24 %]
		CYP19A1	210 [3.96 %]			EGF	97 [5.26 %]
		SLC20A2	159 [3 %]			CDKN2A	90 [4.88 %]
		CEACAM3	151 [2.85 %]			SLC20A2	82 [4.45 %]
		BCL2L14	129 [2.43 %]			ABCB1	81 [4.39 %]
		ABCB1	129 [2.43 %]			BCL2L14	69 [3.74 %]
Italy	4621	ERBB2	808 [17.49 %]	Australia	1715	ESR1	260 [15.16 %]
		ESR1	727 [15.73 %]			ERBB2	166 [9.68 %]
		PGR	404 [8.74 %]			PGR	123 [7.17 %]
		EGF	298 [6.45 %]			BRCA1	120 [7 %]
		CDKN2A	297 [6.43 %]			EGF	94 [5.48 %]
		SLC20A2	238 [5.15 %]			SLC20A2	85 [4.96 %]
		BRCA1	197 [4.26 %]			BRCA2	73 [4.26 %]
		INS	171 [3.7 %]			INS	72 [4.2 %]
		TKT	159 [3.44 %]			ARL11	71 [4.14 %]
		CYP19A1	156 [3.38 %]			CDKN2A	68 [3.97 %]

## Footnotes

1 http://www.knime.org (last visited 24 Nov 2014).

2 http://gephi.github.io (last visited 24 Nov 2014).

3 https://developers.google.com/maps/ (last visited 24 Nov 2014).

4 http://www.genemania.org (last visited 24 Nov 2014).

5 http://thebiogrid.org (last visited 24 Nov 2014).

6 http://www.disgenet.org/web/DisGeNET/v2.1 (last visited 24 Nov 2014).

7 http://django.nubic.northwestern.edu/fundo (last visited 24 Nov 2014).

8 http://www.mathworks.com/matlabcentral/fileexchange/7486-clustering-toolbox (last visited 24 Nov 2014).

9 http://ghr.nlm.nih.gov/gene/CDH1 (last visited 24 Nov 2014).

10 http://www.ncbi.nlm.nih.gov/gene/5602 (last visited 24 Nov 2014).
